# Targeting K_Ca_3.1 channels to overcome erlotinib resistance in non-small cell lung cancer cells

**DOI:** 10.1038/s41420-023-01776-5

**Published:** 2024-01-04

**Authors:** Luca Matteo Todesca, Matthias Gerke, Emma Etmar Bulk, Magdalena Bachmann, Alisa Rudersdorf, Lorenzo Antonuzzo, Serena Pillozzi, Martina Düfer, Ildiko Szabo, Albrecht Schwab

**Affiliations:** 1 Institute of Physiology II, University of Münster, Münster, Germany; 2 Department of Biology, University of Padova, Padua, Italy; 3 Institute of Pharmaceutical and Medicinal Chemistry, University of Münster, Münster, Germany; 4 Department of Experimental and Clinical Medicine, University of Florence, Florence, Italy

**Keywords:** Non-small-cell lung cancer, Non-small-cell lung cancer

## Abstract

Almost all non-small cell lung cancer (NSCLC) patients initially responding to EGFR tyrosine kinase inhibitors (TKIs) develop acquired resistance. Since K_Ca_3.1 channels, expressed in mitochondria and plasma membrane, regulate similar behavioral traits of NSCLC cells as EGFR, we hypothesized that their blockade contributes to overcoming EGFR-TKI resistance. Meta-analysis of microarray data revealed that K_Ca_3.1 channel expression in erlotinib-resistant NSCLC cells correlates with that of genes of integrin and apoptosis pathways. Using erlotinib-sensitive and –resistant NSCLC cells we monitored the role of mitochondrial K_Ca_3.1 channels in integrin signaling by studying cell-matrix adhesion with single-cell force spectroscopy. Apoptosis was quantified with fluorescence-based assays. The function of mitochondrial K_Ca_3.1 channels in these processes was assessed by measuring the mitochondrial membrane potential and by quantifying ROS production. Functional assays were supplemented by biochemical analyses. We show that K_Ca_3.1 channel inhibition with senicapoc in erlotinib-resistant NSCLC cells increases cell adhesion by increasing β1-integrin expression, that in turn depends on mitochondrial ROS release. Increased adhesion impairs migration of NSCLC cells in a 3D matrix. At the same time, the senicapoc-dependent ROS production induces cytochrome C release and triggers apoptosis of erlotinib-resistant NSCLC cells. Thus, K_Ca_3.1 channel blockade overcomes EGFR-TKI resistance by inhibiting NSCLC motility and inducing apoptosis.

## Introduction

Lung cancer is the second most common type of cancer and a leading cause of cancer-related death with non-small cell lung cancer (NSCLC) being the predominant subtype (85%) [1, 2]. For this subtype the patients’ 5-year survival rate reaches only 5% [3]. Treatment options for the advanced stages vary from external radiation therapy to combination chemotherapy and targeted therapy, accomplished according to the respective molecular classification.

The EGF receptor (EGFR) is one of the most important proto-oncogenes in this context [4]. EGFR is overexpressed in more than 60% of all NSCLCs and regulates several cellular processes relevant for tumor progression [5]. NSCLC frequently harbors activating EGFR mutations, usually located between exon 18 and 21, leading to its constitutive activation [6]. Therefore, the EGFR tyrosine kinase inhibitors (EGFR-TKI) such as erlotinib became important therapeutics that are commonly utilized in patients with metastatic NSCLC [7]. Unfortunately, while most EGFR-mutant NSCLCs initially respond to EGFR-TKIs, they ultimately become resistant resulting in tumor relapse [8]. The most common mechanism causing EGFR-TKI resistance is the development of secondary mutations, such as the T790M mutation. It accounts for 50–60% of cases with acquired resistance to first generation EGFR-TKIs (e.g. gefitinib or erlotinib) [9, 10]. The development of new generation EGFR-TKIs, such as osimertinib, partially solved the problem. However, also in this case acquired resistance is recurrent during patient treatment [11].

Thus, there is still an unmet need for improved therapeutic concepts for the treatment of NSCLC. A promising novel approach is targeting ion channels in cancer. Several studies showed that the combination of first-line treatments with ion channel modulators affects “conventional” cancer treatment by lowering doses of the chemotherapeutic drugs thereby reducing undesirable side effects [12]. In this context, K_Ca_3.1 is a particularly attractive target. K_Ca_3.1 has multiple roles in NSCLC [13]. Its expression has prognostic value [14]. Moreover, it contributes to disease progression. In vitro and in vivo experiments, previously performed by our group, showed that K_Ca_3.1 silencing or inhibition with the K_Ca_3.1 blockers senicapoc or TRAM-34 impair migration, invasion, proliferation and tumor growth [15, 16]. Moreover, the combination with the K_Ca_3.1 channel blocker senicapoc increases the sensitivity to erlotinib, especially under hypoxic conditions [17]. K_Ca_3.1 is also expressed in mitochondria of NSCLC cells where it regulates the mitochondrial membrane potential and thereby the mitochondrial production of superoxide [18]. Here, we unraveled mechanisms by which K_Ca_3.1 channels located in the inner membrane of mitochondria contribute to EGFR-TKI resistance of NSCLC cells.

## Results

### Altered K_Ca_3.1 channel expression is linked to dysregulation of several pathways

**Fig. 1:**
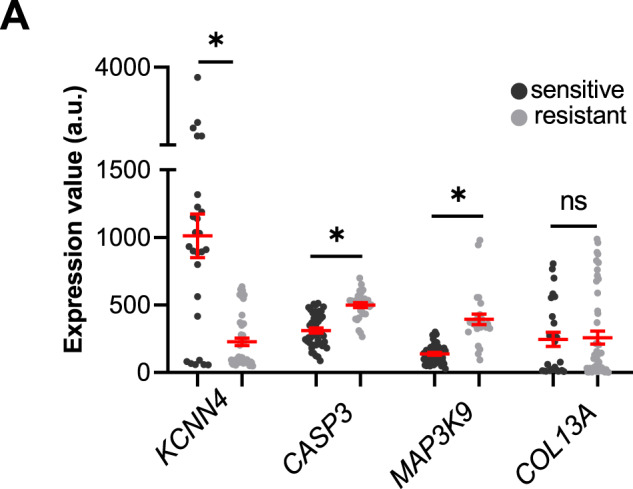
Genes highly correlated with the altered expression of K_Ca_3.1 A. Each point represents the gene expression value (estimated number of RNA transcripts) for a cell line represented by the GSE31625, GSE38310 and GSE38404 microarray datasets. The gene expression values have been normalized by removing non-biological variation, and generating the final probe set expression values. GSE31625 gene expression was evaluated with MAS5 normalization, GSE38310 and GSE38404 were evaluated with RMA normalization. *p < 0.05. For the statistical analysis, a two-tailed Student’s *t*-test was performed.

Performing a Spearman correlation analysis within the NSCLC cells datasets GSE38310, GSE38404 and GSE3125, we found that the expression of *CASP3* (caspase 3) and *MAP3K9* (mitogen-activated protein kinase 9) inversely correlated with that of *KCNN4*. *MAP3K9* is recognized as part of the JNK pathway and its function is mainly related to apoptosis by inducing the caspase cascade [19]. As shown in Fig. 1, these two genes, *MAP3K9* and *CASP3*, are expressed at higher levels in erlotinib-resistant than erlotinib-sensitive cells (394.6 ± 39.1 a.u. vs 137.4 ± 10.0 a.u. and 497.5 ± 18.3 a.u. vs 310.6 ± 19.1 a.u., respectively). We showed earlier that K_Ca_3.1 expression is lower in erlotinib-resistant cells [17]. *COL13A1* (collagen type XIII α1 chain; involved in the integrin pathway and linked to the activation of β1-integrin [20]) expression levels are variable in the three data sets. While there is a correlation between *COL13A1* and *KCNN4* expression in the individual data sets, the large scatter of expression values dissipates this correlation when combining all three data sets. Both the integrin and apoptosis pathways are among the top 10 of the most represented pathways related to the 433 differentially expressed genes (DEGs) between erlotinib-resistant and -sensitive cells. Of the 433 DEGs, 7 are part of the integrin pathway and 11 of the apoptosis pathway.

### Caspase 3 activity increases after senicapoc treatment of NSCLC cells

**Fig. 2:**
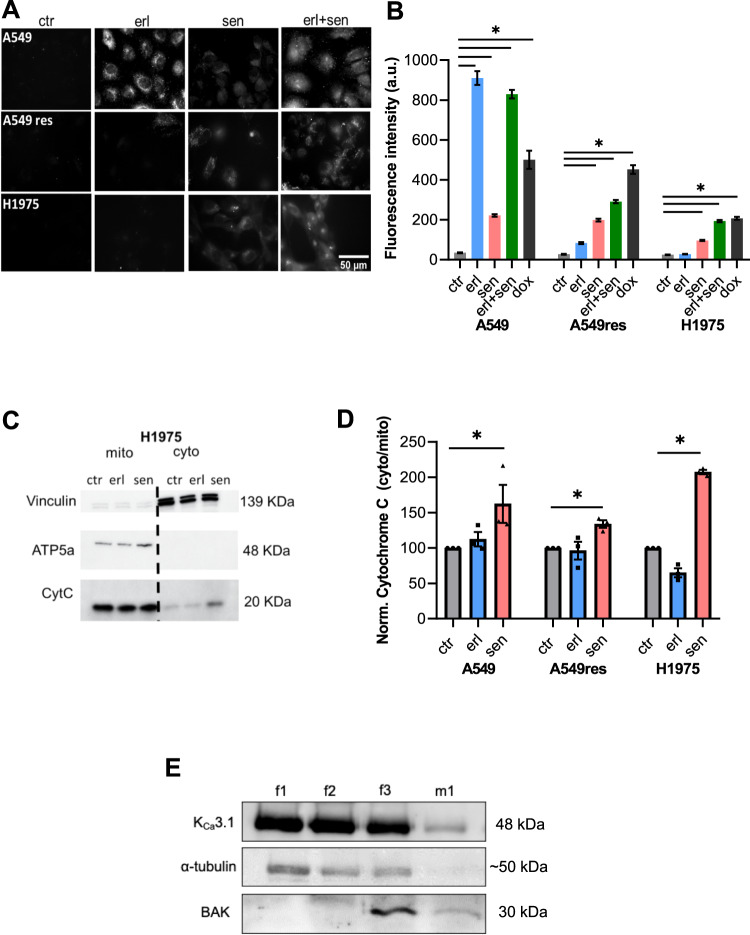
Combining the K_Ca_3.1 blocker senicapoc with erlotinib induces apoptosis in erlotinib-resistant NSCLC cells A. **A** Represenative images of the 3 NSCLC cell lines stained with Red-DEVD-FMK under control conditions (DMSO 1:1000), after treatment with erlotinib (10 µM), senicapoc (30 µM) and the combination of erlotinib (10 µM) and senicapoc (30 µM). **B** Summary of the caspase 3 activation results of the 3 cell lines under 5 different conditions (ctr = DMSO 1:1000; erl erlotinib 10 µM, erl + sen erlotinib 10 µM + senicapoc 30 µM, sen senicapoc 30 µM, dox doxorubicin 100 nM) (*N* = 4; *n* ≥ 250). The bar plots represent the mean cytoplasmic fluorescence intensity of the cells after 24 h treatment. For each N cells from at least 10 visual fields with at least 6 cells were analyzed. *: *p* < 0.05, one-way ANOVA with Tukey’s posthoc test. **C** Western blots used to quantify cytochrome C release into the cytosol of H1975 cells. Vinculin (139 kDa) and ATP5a (48 kDa) served as cytosolic and mitochondrial markers, respectively. **D** Comparison of the relative ratio between cytoplasmatic cytochrome C and mitochondrial cytochrome C after treatment with different compounds (ctr = DMSO 1:1000; erl erlotinib 10 µM, sen senicapoc 30 µM). *p < 0.05; a one-way ANOVA with Tukey’s posthoc test (*N* = 3). **E** K_Ca_3.1 expression in mitochondria from NSCLC patient samples. f1: whole-cell extract; f2: membrane-enriched fraction; f3: mitochondria-enriched fraction; m1: Percoll-purified mitochondrial fractions. Cytosolic marker α-tubulin and mitochondrial membrane marker BAK are also shown.

Based on the results of the bioinformatic analyses we studied the involvement of K_Ca_3.1 channels in apoptosis of erlotinib-sensitive and -resistant NSCLC cells using DEVD-FMK conjugated to sulfo-rhodamine as an in-situ marker. Doxorubicin was used as positive control [21]. Figure 2A displays the fluorescence of the apoptosis marker in the NSCLC cells. As expected sensitive A549 cells show a strong activation of caspase 3 after erlotinib treatment (910.0 ± 34.2 a.u. vs 35.0 ± 2.10 a.u. under control conditions). In partially erlotinib-resistant A549res cells, erlotinib treatment induces a much smaller increase of caspase 3 activation (83.7 ± 5.0 a.u. vs 27.1 ± 2.0 a.u.). In the fully resistant H1975 cells erlotinib has no effect on caspase 3 activation (24.7 ± 1.0 a.u. control vs 27.9 ± 1.1 a.u. erlotinib; Fig. 2B).

In all three cell lines senicapoc alone increases caspase 3 activation (222.2 ± 6.4 a.u. in A549; 198.7 ± 6.5a.u. in A549res; 96.3 ± 3.1 a.u. in H1975). The double treatment showed not only a higher caspase 3 activation compared to control in A549 cells (829.6 ± 21.1 a.u.) but also a noticeably higher caspase 3 activation compared to single treatments of A549res cells with erlotinib or senicapoc (A549res: 291.3 ± 7.9 a.u.). This also applies to the fully resistant H1975 cells. Caspase 3 activation is twice as high following the double treatment than with senicapoc alone (193.3 ± 5.9 a.u.). The following experiments are aimed at elucidating mechanisms by which K_Ca_3.1 channel inhibition apparently overcomes erlotinib resistance.

### Cytochrome C is released into the cytosol of senicapoc-treated NSCLC cells

Cytochrome C is one of the main activators of the intrinsic apoptosis pathway [22]. We therefore tested whether K_Ca_3.1 inhibition induces cytochrome C release into the cytosol after 6 h treatment. Cytochrome C was normalized to vinculin and ATP5a as housekeeping proteins for the cytoplasmic and the mitochondrial fractions, respectively (see Fig. 2C and Supplementary Fig. 1A). These experiments are summarized in Fig. 2D. Cytochrome C release into the cytoplasm rises by ~60%, ~30% and ~100% after senicapoc treatment of A549, A549res and H1975 cells, respectively. This points to a potential role of mitochondrial K_Ca_3.1 channel activity in apoptosis induction.

### K_Ca_3.1 is expressed in mitochondria of surgical tumor samples from NSCLC patients

We recently showed the expression of K_Ca_3.1 channels in mitochondria of the NSCLC cell lines A549, H1299, and H1975 [18]. Here we applied Western blotting to verify that K_Ca_3.1 is also expressed in the mitochondria of primary NSCLC cells (adenocarcinoma) derived from surgical patient samples. We used BAK and α-tubulin as loading controls for mitochondrial and whole cell lysates, respectively. Figure 2E shows that the channel is present in the mitochondrial fraction (“m1”) of the patient samples that also contained BAK. In contrast, cytosolic α-tubulin is almost absent in this fraction.

### K_Ca_3.1 channel-dependent dynamics of the mitochondrial membrane potential in erlotinib-sensitive and –resistant NSCLC cell lines

**Fig. 3:**
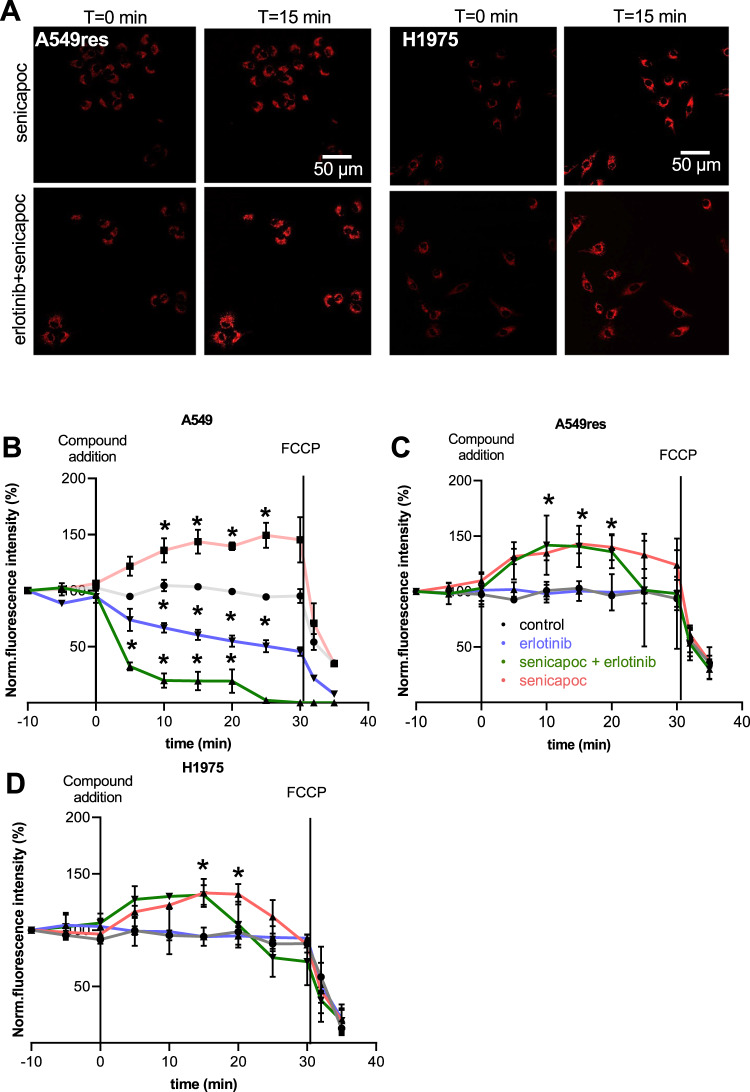
K_Ca_3.1 channels control the mitochondrial membrane potential in NSCLC cells. **A** Representative images of NSCLC cells stained with TMRM at different time points after treatment with DMSO (control), senicapoc (30 µM), erlotinib in combination with senicapoc (10 µM + 30 µM). **B**–**D**. Mean values of the TMRM fluorescence intensity normalized to baseline values at *t* = 0. The assays were performed on 3 NSCLC cell lines under 4 different conditions (*N* = 3; *n* ≥ 25) (ctr = DMSO 1:1000; erl erlotinib 10 µM; erl+sen erlotinib 10 µM + senicapoc 30 µM; sen senicapoc 30 µM). Compounds were added at *t* = 0. After 30 min of recording FCCP (1 µM) was added to induce a complete depolarization of the mitochondrial membrane potential. *p < 0.05; a two-way ANOVA with Tukey’s posthoc test. For comparison, B contains values of the mitochondrial membrane potential from Bulk 2022 (control, senicapoc).

After showing the mitochondrial expression of K_Ca_3.1 channels in NSCLC cells, we tested their impact on the membrane potential of the inner mitochondrial membrane by using the cationic fluorescent dye Tetramethylrhodamine Methyl Ester (TMRM) (Fig. 3A). These experiments extend our previous observations [18] by also investigating the effect of erlotinib and its combination with senicapoc (Fig. 3). In contrast to senicapoc, which we had shown to increase TMRM fluorescence by 49.3 ± 11.2% [18], erlotinib elicits a TMRM fluorescence intensity decrease by 33.1 ± 4.3% within 10 min corresponding to a depolarization of the mitochondrial membrane potential of A549 cells. The double treatment with senicapoc and erlotinib induces an even more pronounced depolarization: -67.7 ± 3.5% of TMRM fluorescence intensity within 5 min (Fig. 3B).

As expected erlotinib does not change the mitochondrial membrane potential of A549res and H1975 cells, while senicapoc induces an increase of the TMRM fluorescence (corresponding to the expected hyperpolarization) by 34.7 ± 6.6% within 10 min in A549res cells and 22.2 ± 0.8% in H1975 cells (Fig. 3C, D). Similarly, the double treatment with senicapoc and erlotinib results initially in increased fluorescence within 10 min: +27.6 ± 9.7% in A549res cells and +22.2 ± 0.8% in H1975 cells. Notably, the hyperpolarization is only transient in erlotinib-resistant cells. It is followed by a depolarization. TMRM fluorescence drops by 28.7% in A549res and by 13.5% in H1975 at *t* = 25 min.

### Senicapoc in combination with erlotinib induces ROS release in erlotinib-resistant NSCLC cells

**Fig. 4:**
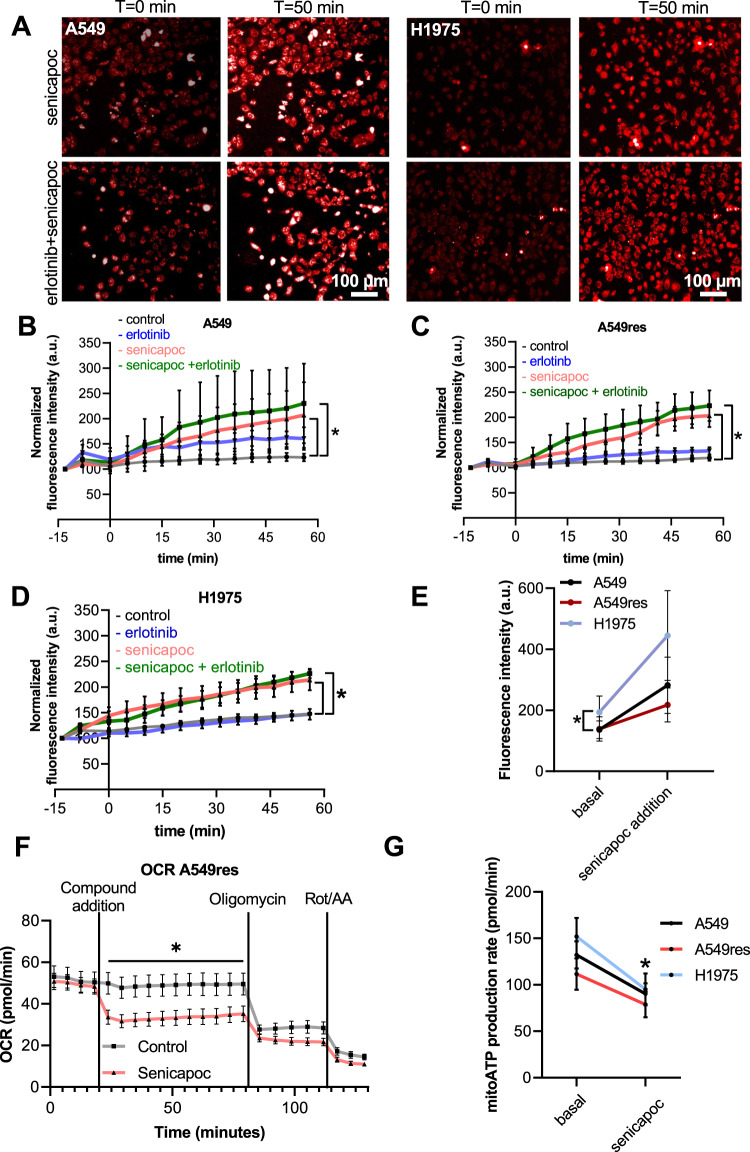
K_Ca_3.1 inhibition with senicapoc triggers superoxide generation and reduces mitochondrial oxygen consumption in NSCLC cells. **A** Representative images of A549 and H1975 cells stained with MitoSOX and acquired at *t* = 0 min and *t* = 50 min. **B**–**D**. Mean values of the MitoSOX fluorescence intensities normalized to *t* = 0 of the baseline measurements. The assays were performed on 3 NSCLC cell lines under 5 different conditions (*N* = 4) (ctr = DMSO 1:1000; erl erlotinib 10 µM, erl+sen erlotinib 10 µM + senicapoc 30 µM, sen senicapoc 30 µM, antA antimycin A 2 µM). *p < 0.05; a two-way ANOVA with Tukey’s posthoc test. Control and senicapoc conditions have also been performed in Bulk 2022. For comparison, here are repeated with a different setup (Operetta system). **E** Superoxide levels measured under basal conditions (*t* = 0 min) and 56 min after adding senicapoc to the cells (*N* = 4; *n* ≥ 4 for each NSCLC cell line). **F** Oxygen consumption rate (OCR) of A549res cells under control conditions (DMSO 1:1000) and following the application of senicapoc (30 µM). After 79 min oligomycin A is added and after 112 min Rot/AA, a mix of antimycin A (AA) and rotenone (Rot), is added. *p < 0.05; multiple *t*-test between each time point. **G** Mitochondrial ATP production (mitoATP) under basal conditions and following the addition of senicapoc (30 µM; *N* = 4). *p < 0.05; paired *t*-test between basal levels and after senicapoc addition for each cell line.

To check whether K_Ca_3.1-mediated changes of the mitochondrial membrane potential are followed by a ROS release, we used the fluorescent mitochondrial superoxide indicator MitoSOX (Fig. 4). As shown previously ROS release rises in senicapoc-treated A549 cells after ~40 min (fluorescence increase compared to baseline values: control 22.9 ± 4.3% vs senicapoc 88.2 ± 25.4%) as shown previously (Bulk 2022). Following double treatment with erlotinib and senicapoc this occurs after 30 min (18.6 ± 4.9% control vs 102.3 ± 41.3% senicapoc and erlotinib; Fig. 4B).

A549res and H1975 cells show a similar behavior when treated with senicapoc or with senicapoc and erlotinib in combination (Fig. 4C, D). By the end of the measurements, senicapoc or the combination have caused marked increases of the MitoSOX fluorescence intensities (A549res cells: control: 19. ± 5.8%, senicapoc: 103.1 ± 22.5%; double treatment: 123.0 ± 30.5%; H1975 cells: control: 47.3 ± 3.6%, senicapoc: 113.1 ± 20.4%; double treatment: 126.3 ± 9.1%). Treating A549res or H1975 cells with erlotinib alone does not lead to ROS production (A549res cells 33.3 ± 7.3%; H1975 cells: 46.7 ± 10.3%).

Interestingly, H1975 have a different initial ROS levels (Fig. 4E) (234.4 ± 48.0 a.u.) than A549 (136.3 ± 28.6 a.u.) and A549res cells (139.0 ± 39.7 a.u.). This is even more pronounced after senicapoc treatment, when comparing the raw values at *t* = 56 min (end of the experiment): ROS levels reach 445.0 ± 147.1 a.u. in H1975, 281.9 ± 92.2 a.u. in A549 and 217.6 ± 56.0 a.u. in A549res cells.

### K_Ca_3.1 channel inhibition reduces oxygen consumption and mitochondrial ATP production in NSCLC cells

Since the mitochondrial membrane potential provides the driving force for the respiratory chain, we next assessed the effect of K_Ca_3.1 channel inhibition on OCR and ATP production. To this end, we performed a Seahorse XF ATP production assay. As shown in Fig. 4F (and Supplementary 1B &1C), senicapoc leads to a marked drop of the OCR in all three cell lines. OCR (basal levels: A549: 49.8 ± 4.2 pmol/min; A549res: 50.8 ± 3.9 pmol/min; H1975: 63.2 ± 7.2 pmol/min) drops by 30.9%, 37.6% and 31.5%, respectively, after 5–10 min following addition of senicapoc. The senicapoc-induced decrease in the OCR is accompained by a decrease of the mitochondrial ATP production in all the three cell lines. Mitochondrial ATP production decreases by ~40%, ~30% and ~37% after senicapoc treatment of A549, A549res and H1975 cells, respectively (Fig. 4G).

Collectively, our data thus far show that K_Ca_3.1 channels overcome erlotinib resistance of NSCLC cells by their marked impact on the function of mitochondria.

### K_Ca_3.1 blockade increases NSCLC cell adhesion to and inhibits migration in an ECM-like matrix

**Fig. 5:**
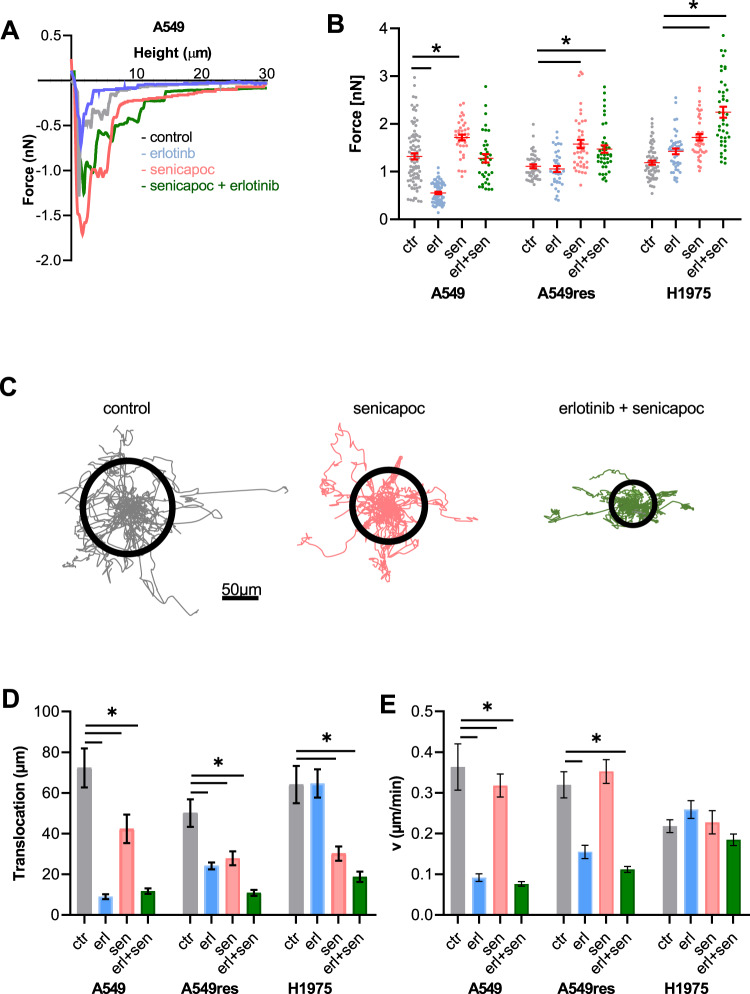
Inhibition of K_Ca_3.1 channels impairs NSCLC cell-matrix and migration in a 3D matrix. **A** Original force-distance curves of A549 cells under control conditions and in the presence of erlotinib and/or senicapoc (24 h pretreatment). **B** Summary of single-cell force spectroscopy experiments in A549, A549res and H1975 (*N* = 7, *n* = 35). Each dot represents an interaction between the cancer cell and the ECM-like matrix. 4 different conditions were evaluated (ctr = DMSO 1:1000; erl erlotinib 10 µM, erl + sen erlotinib 10 µM + senicapoc 30 µM, sen senicapoc 30 µM). *p < 0.05; one-way ANOVA with Tukey’s posthoc test. **C** Trajectories of migrating A549 cells normalized to common starting point. Migration was recorded under control conditions, after senicapoc treatment (30 µM) or in the combined presence of erlotinib (10 µM) and senicapoc (30 µM). Cell paths are normalized to common starting points and the radii of the black circles represent the mean translocation covered during the course of the experiments. **D**, **E** Summary of the experiments shown in C. Mean translocation and speed (*N* = 3; *n* = 30). *p < 0.05; one-way ANOVA with Tukey’s posthoc test.

The bioinformatic analysis revealed a correlation between the expression of K_Ca_3.1 and genes involved in the integrin pathway. Therefore, we explored the impact of K_Ca_3.1 modulation on integrin signaling in erlotinib-sensitive and -resistant NSCLC cells. This was mainly done by quantifying the maximal adhesion force of NSCLC cells to an ECM-like matrix with single-cell force spectroscopy. In Fig. 5A, B we show measurements with A549 cells: Under control conditions (1:1000 DMSO) the cells have a maximal adhesion force of 1.31 ± 0.06 nN. Adhesion force strongly increases to 1.71 ± 0.06 nN when the K_Ca_3.1 channel is inhibited with senicapoc. When cells are treated with erlotinib cell adhesion to the matrix decreases to 0.55 ± 0.02 nN. The double treatment with senicapoc and erlotinib restores the maximal adhesion force to control levels (1.28 ± 0.06 nN).

As expected erlotinib has no impact on the adhesion of A549res and H1975 cells to the ECM matrix (A549res cells: control: 1.11 ± 0.04 nN, erlotinib: 1.06 ± 0.06 nN; H1975 cells: control: 1.19 ± 0.05 nN, erlotinib: 1.41 ± 0.06 nN; Fig. 5B). Senicapoc leads to an increase of the adhesion force (A549res cells: 1.47 ± 0.08 nN; H1975 cells: 1.72 ± 0.07 nN). This increase becomes even more evident in H1975 cells treated with senicapoc and erlotinib, where adhesion force markedly rise (2.24 ± 0.11 nN). In A549res cells the double treatment induces a higher adhesion force, comparable to the one observed with senicapoc alone (1.47 ± 0.08 nN).

We next tested whether the K_Ca_3.1-dependent regulation of cell-matrix adhesion of (erlotinib-resistant) NSCLC cells modulates cell migration in a three-dimensional ECM-like collagen matrix. Figure 5C illustrates the trajectories of the A549 cells that are normalized to common starting points. Figure 5D, E summarize the results of the migration experiments. Under control conditions (1:1000 DMSO) A549 cells have a migration speed of 0.36 ± 0.06 μm/min and the translocation amounts to 72.3 ± 9.6 μm. Inhibiting K_Ca_3.1 channels with senicapoc reduces their translocation (42.3 ± 7.0 μm), but does not affect the migration speed (0.32 ± 0.03 μm/min). Erlotinib or the combination of erlotinib and senicapoc drastically decrease both parameters (migration speed: 0.09 ± 0.01 μm/s and 0.08 ± 0.06 μm/s, respectively, translocation: 9.0 ± 1.2 μm and 11.7 ± 1.3 μm, respectively).

In A549res cells, senicapoc reduces the translocation by ~45% (50.1 ± 6.7 μm vs 27.8 ± 3.4 μm), while the migration speed remains unchanged (0.32 ± 0.03 μm/min vs 0.35 ± 0.03 μm/min). H1975 cells respond in a similar way (translocation: 64.1 ± 9.2 μm vs 30.2 ± 3.5 μm; speed: 0.22 ± 0.02 μm/min vs 0.23 ± 0.03 μm/min).

Since A549res are partially erlotinib-resistant, the effect of erlotinib is still present but smaller than in A549 cells. Translocation and migration speed decrease to 24.8 ± 3.4 μm and 0.16 ± 0.02 μm/min, respectively. Erlotinib does not affect translocation and migration speed of H1975 cells (translocation: 64.1 ± 9.2 μm vs 64.7 ± 7.0 μm; migration speed: 0.21 ± 0.02 μm/min vs 0.26 ± 0.02 μm/min).

In the combined presence of senicapoc and erlotinib the translocation of the A549res cells is reduced even more to 10.9 ± 1.5 μm. The same effect on translocation is visible in H1975 cells (18.8 ± 2.5 μm). The migration speed of H1975 cells is not affected by the double treatment (0.18 ± 0.01 μm/min). The differential effect of senicapoc or its combination with erlotinib on translocation and migration speed can be interpreted as “running on the spot”. This effect is particularly important in erlotinib-resistant cells. Thus, cell-matrix adhesion and thereby cell migration are further behavioral traits of NSCLC cells where erlotinib-resistance can be overcome by K_Ca_3.1 channel inhibition. The following experiments aim at determining the potential mechanism underlying the altered migratory behavior.

### Senicapoc and its combination with erlotinib increase adhesion to the ECM by elevating β1-integrin expression in a ROS-dependent way

**Fig. 6:**
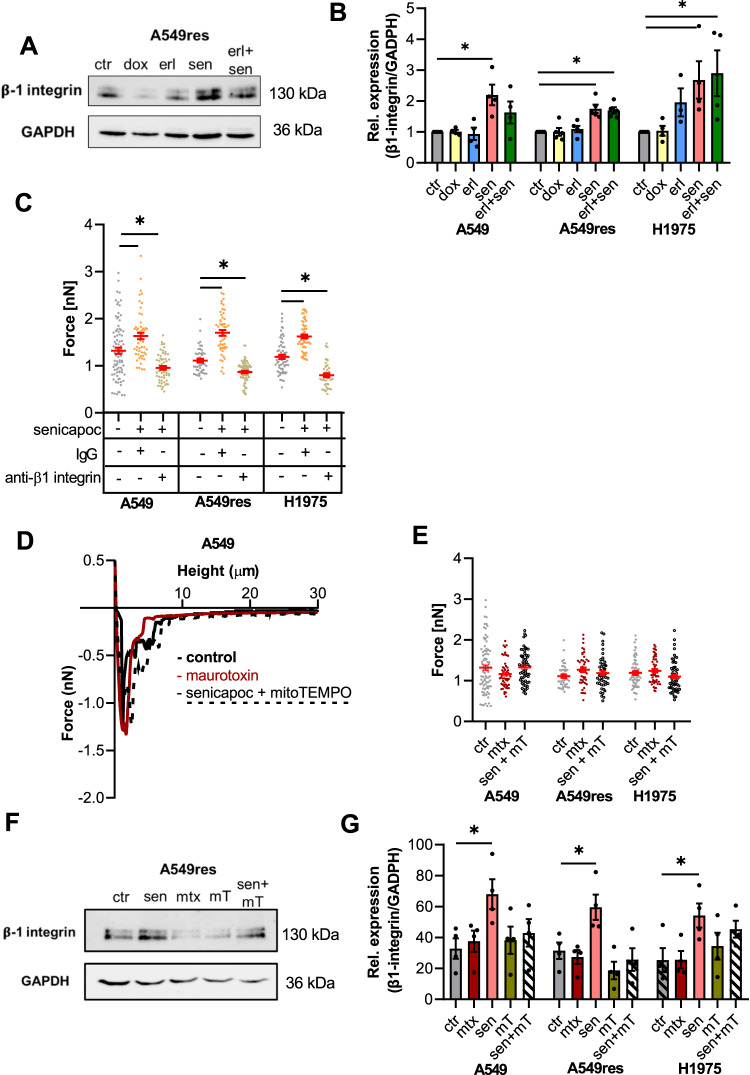
Senicapoc-dependent ROS release accounts for increased β1-integrin expression and cell-matrix adhesion in NSCLC cells. **A** Western blots showing the regulation of β1-integrin expression in A549res. Protein lysates were obtained after 24 h pretreatment. **B** Quantification of β1-integrin expression normalized to GAPDH (*N* = 4). (ctr;= DMSO 1:1000; erl erlotinib 10 µM, erl+sen erlotinib 10 µM + senicapoc 30 µM, sen senicapoc 30 µM, dox doxorubicin 100 nM). *p < 0.05; Mann–Whitney *U*-test. **C** Summary of single-cell force spectroscopy experiments in the presence of a blocking β1-integrin antibody (10 ng/µL) and an unspecific IgG as control after senicapoc pre-treatment for 24 h (*N* = 7 for each condition). Each dot represents one single interaction between a cancer cell and the ECM-like matrix. *: *p* < 0.05; one-way ANOVA with Tukey’s posthoc test. **D** Original force-distance curves of A549 cells under control conditions and in the presence of maurotoxin or senicapoc in combination with mitoTEMPO (24 h pre-treatment). **E** Summary of adhesion force measurements. Each dot represents one single interaction between a cancer cell and the ECM-like matrix (mtx maurotoxin 20 nM), sen+mT combination of senicapoc and mitoTEMPO (30 μM + 2μM) and DMSO 1:1000 as negative control (ctr). (*N* = 7, *n* = 35). *: *p* < 0.05; one-way ANOVA with Tukey’s posthoc test. **F** Standard techniques for Western blotting were performed on A549res cells in 5 different conditions. Protein lysates were obtained after 24 h pretreatment. **G** Relative quantification of β1-integrin normalized to GAPDH (*N* = 4) in 5 different conditions (sen senicapoc 30 μM), mtx maurotoxin 20 nM, mT mitoTEMPO 2 μM, sen + mT combination of senicapoc and mitoTEMPO (30 μM + 2μM) and DMSO 1:1000 as control (ctr). The symbol * denotes a *p* < 0.05. For the statistical analysis, a Mann–Whitney *U*-test was performed.

We tested whether β1-integrin expression is involved in the K_Ca_3.1 channel-dependent increase of adhesion between NSCLC cells and the ECM. Western blotting indicated that a 24 h treatment of A549, A549res and H1975 cells with senicapoc alone increases the expression of β1-integrin (Fig. 6A, B; Supplementary Fig. 2A). The same applies for the double treatment of the two resistant cell lines.

The functional relevance of this observation was tested with single cell force spectroscopy experiments. NSCLC cells were preincubated with a blocking β1-integrin antibody or with an IgG control antibody. As shown in Fig. 6C, the senicapoc-induced increase of the adhesion force is prevented by the β1-integrin blocking antibody, but not by an IgG control antibody. The respective values are: A549 cells: 1.31 ± 0.06 nN (control), 0.96 ± 0.03 nN (β1-integrin blocking antibody plus senicapoc), 1.63 ± 0.04 nN (IgG control antibody plus senicapoc). A549res cells: 1.11 ± 0.04 nN (control), 0.87 ± 0.03 nN (β1-integrin blocking antibody plus senicapoc), 1.70 ± 0.06 nN (IgG control antibody plus senicapoc). The same applies for H1975 cells, where the adhesion in the presence of the β1-integrin blocking antibody amounts to 0.80 ± 0.04 nN (control value 1.19 ± 0.04 nN). These results suggest that the K_Ca_3.1 channel-dependent increase of cell-matrix adhesion of NSCLC cells and their migratory phenotype are mediated at least in part by increased β1-integrin expression.

Finally, we tested whether K_Ca_3.1 channels located in the plasma membrane or in the inner membrane of mitochondria are required for regulating cell-matrix adhesion and β1-integrin expression. NSCLC cells were treated with the small peptide toxin maurotoxin (mtx) that cannot pass the plasma membrane and therefore does not inhibit mitochondrial K_Ca_3.1 channels. Alternatively, we used the ROS scavenger mitoTEMPO in combination with senicapoc. Figure 6D & E show that maurotoxin does not change the maximal adhesion force in A549 cells (1.31 ± 0.06 nN (mtx) vs 1.16 ± 0.04 nN (ctr)). Moreover, the senicapoc-induced increased adhesion is abolished when A549 cells are treated with the ROS scavenger mitoTEMPO in combination with senicapoc (1.33 ± 0.05 nN).

We obtained very similar results with the two erlotinib-resistant cell lines: Maurotoxin does not increase the maximal adhesion force in A549res cells (1.26 ± 0.06 nN (mtx) vs 1.11 ± 0.04 nN (ctr)) and in H1975 cells (1.24 ± 0.04 nN (mtx) vs 1.19 ± 0.05 nN (ctrl)) (Fig. 6E). Moreover, mitoTEMPO prevents the senicapoc-induced rise of the maximal adhesion force in A549res cells (1.71 ± 0.04 nN) (senicapoc) vs 1.10 ± 0.05 nN (senicapoc + mitoTEMPO) and in H1975 cells (1.58 ± 0.09 nN (senicapoc) vs 1.19 ± 0.06 nN (senicapoc + mitoTEMPO)).

Adhesion force measurements were complemented by determining the expression of β1-integrin under the same experimental conditions. Figure 6F & G (see also Supplementary 2B) reveal that the senicapoc-dependent increase of β1-integrin expression is prevented in the presence of mitoTEMPO and is not present when cells are treated with maurotoxin. These results indicate that the higher adhesion evoked by senicapoc treatment is mainly due to its inhibitory function on mitoK_Ca_3.1 channels.

### Senicapoc-dependent β1-integrin expression is regulated by activation of the JNK pathway

**Fig. 7:**
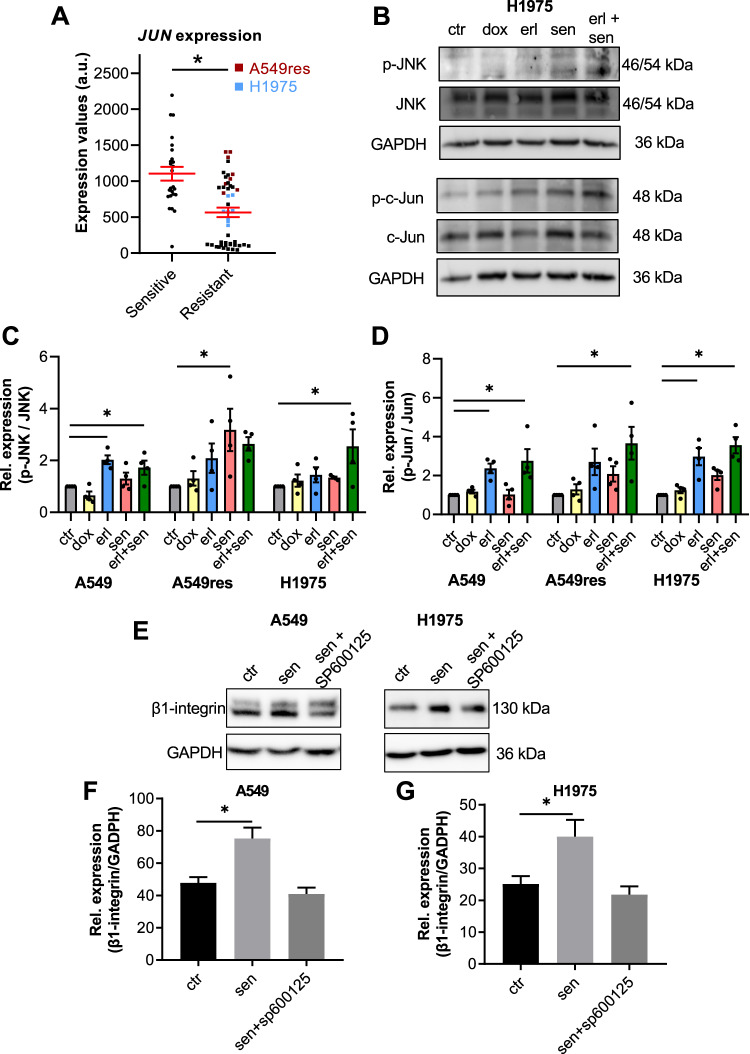
JNK activation regulates β1-integrin expression in NSCLC cell lines. **A** Comparison of gene expression values of JUN. Each point represents the gene expression value (estimated number of RNA transcripts) for each cell line represented by the GSE31625, GSE38310 and GSE38404 microarray datasets. **B** Western blots to illustrate phosphorylation of Jun and JNK in H1975 cells under 5 different conditions. Protein lysates were obtained after 24 h pretreatment. **C**, **D** Relative quantification of p-JNK and p-Jun normalized to total JNK and total c-Jun expression, respectively (*N* = 4) (ctr = DMSO 1:1000; erl erlotinib 10 µM, erl+sen erlotinib 10 µM + senicapoc 30 µM, sen senicapoc 30 µM, dox doxorubicin 100 nM). *p < 0.05; Mann–Whitney *U*-test. **E** Western blots to show the effect of JNK inhibition with SP600125 (30 µM) on β1-integrin expression. Protein lysates were obtained after 24 h pretreatment. **F** Relative quantification of β1-integrin expression normalized to GAPDH (*N* = 4) (DMSO 1:1000 as control (ctr), sen senicapoc 30 µM, sen + sp600125 senicapoc 30 µM + SP600125 30 µM). *p < 0.05; Mann–Whitney *U*-test.

Finally, we tested whether the JNK pathway is an intermediary between senicapoc-induced mitochondrial ROS release and increase of β1-integrin expression since the bioinformatic analysis revealed a lower expression of one of the JNK pathway proteins, JUN (Jun proto-oncogene, AP-1 transcription factor subunit; Fig. 7A). In all the three cell lines, the double treatment (senicapoc plus erlotinib) causes a JNK and Jun activation as revealed by a higher phosphorylation levels (Fig. 7B-D & Supplementary 2C).

Therefore, we performed another series of Western blot experiments to determine whether blocking JNK activation with the JNK inhibitor SP600125 (MedChemExpress) [23] prevents the β1-integrin up-regulation caused by senicapoc. Figure 7E–F reveal that this is indeed the case. In A549 and H1975 cells the senicapoc-dependent β1-integrin increase is prevented in the presence of SP600125.

## Discussion

This study aimed to address the role of K_Ca_3.1 channels in EGFR-TKI resistance in NSCLC. Growth factor receptors like EGFR and ion channels often functionally cooperate in cancer cells so that anti-cancer effects of TKIs can be potentiated by co-treatment with ion channel modulators [24, 25]. Indeed, frequently therapy resistance is related to overexpression or reduction of K^+^ channel expression [26]. Here we tested whether the efficacy of the EGFR-TKI erlotinib can be enhanced and resistance of NSCLC cells to this drug be overcome by combining it with the highly specific K_Ca_3.1 inhibitor senicapoc. The key results obtained are: (1) K_Ca_3.1 expression is reduced in erlotinib-resistant cells and this is linked to dysregulation of the integrin and apoptosis pathway in resistant cells. (2) Senicapoc co-treatment with erlotinib contributes to overcoming erlotinib-resistance by inducing apoptosis and impairing cancer cell migration via up-regulation of β1-integrin expression. (3) Inhibition of mitochondrial K_Ca_3.1 channels is the central mechanism for overcoming erlotinib resistance, by alterating mitochondrial membrane potential dynamics and ROS release.

The lower expression of K_Ca_3.1 channels in erlotinib-resistant cells can be explained by a lower expression of *JUN* since it is part of the AP-1 complex that regulates K_Ca_3.1 expression [27, 28]. Moreover, c-Jun increases expression and regulates function of the DNA methyltransferase-1 (DNMT1) in cancer cells [29, 30]. Hence, it could also decrease the expression of K_Ca_3.1 channels by enhancing DNA methylation of its promoter [15]. In our case the direct regulation of Jun in K_Ca_3.1 expression results to be the main explanation for a reduction of the channel in erlotinib-resistant cells.

Following the bioinformatic analysis, our functional assays showed that the sensitivity to erlotinib was recovered in A549res and H1975 cells when they were treated in combination with senicapoc. Double treatment induces a marked increase of apoptosis, and at the same time translocation of erlotinib-resistant cells is reduced by 50–60%. The common denominator of both effects is the inhibition of mitochondrial K_Ca_3.1 channels in erlotinib-resistant NSCLC cells. Here we showed for the first time that K_Ca_3.1 channels are also expressed in mitochondria of tissue samples from NSCLC patients. As expected erlotinib does not affect the mitochondrial homeostasis in erlotinib-resistant NSCLC cells. However, the combined application of erlotinib and senicapoc elicits a complex dynamics of the mitochondrial membrane potential. It induces a transient hyperpolarization, which is followed by a depolarization (- occurring after ~20 min of treatment -) and then leads to increased ROS release after ~30 min of treatment. Enhanced mitochondrial ROS production, not apparent in case of erlotinib treatment of A549res and H1975, is therefore an important event to overcome erlotinib resistance upon K_Ca_3.1 channel inhibition.

A sustained ROS generation can trigger the mPTP opening which leads to a depolarization of the mitochondrial membrane potential and a higher release of pro-apoptotic factors from mitochondria [31–33]. Notably, there is a close correlation between ROS production in A549, A549res and H1975 cells after senicapoc treatment (106%, 56% and 130%, respectively) and the increase in the release of pro-apoptotic cytochrome c into the cytoplasm (60%, 30% and 100%, respectively).

ROS production, which includes the activation of the JNK pathway as an intermediary step, is also responsible for an up-regulation of β1-integrin expression. AFM-based single cell force spectroscopy experiments revealed that stimulating the expression of β1-integrins which is particularly important in erlotinib-resistant cells. In these cells the double treatment with senicapoc and erlotinib causes a ROS-dependent up-regulation of the β1-integrin expression and thereby increases adhesion of the cancer cells to the ECM. The movement of cancer cells within a 3D matrix displays a biphasic dependence on adhesion when the environmental properties such as stiffness of the matrix are constant [29]. Hence, increased adhesion results in reduced migration. Acquired resistance also implies a higher invasiveness [34]. Therefore, the combination of erlotinib with senicapoc will decrease the invasiveness of the NSCLC resistant cells and reduce the spreading of the cancer because it impairs migration. In addition to regulating NSCLC cell migration by increasing β1-integrin expression, ROS may also affect migration by directly modulating integrin function [35, 36].

**Fig. 8:**
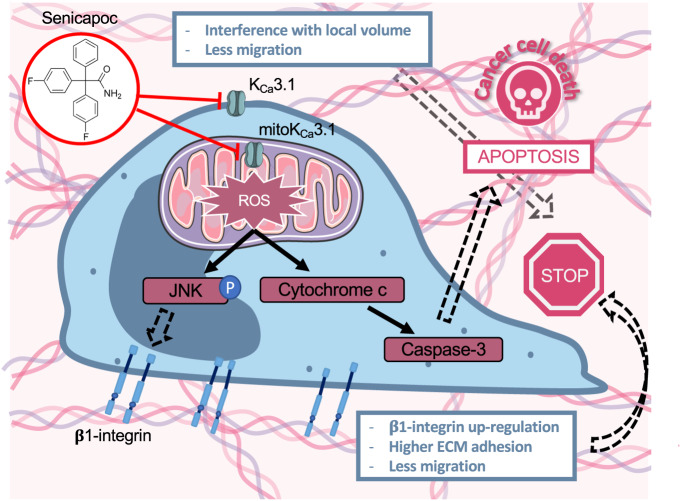
Model of how inhibiting K_Ca_3.1 channels contributes to overcoming erlotinib resistance in NSCLC cells. Senicapoc is acting on two parallel mechanisms: Inhibition of mitoK_Ca_3.1 induces ROS release that leads to apoptosis of cancer cells. At the same time, ROS release is responsible for the increase of β1-integrin expression, increase of adhesion to the ECM and consequently reduction of motility of erlotinib-resistant NSCLC cells. Senicapoc also effecting migration by inhibiting plasma membrane K_Ca_3.1 channel and interfering with local volume changes at the rear end.

Taken together our findings suggest that the combined treatment with erlotinib and senicapoc might lead to a reduction of the tumor size by inducing apoptosis and to less metastatic events by impairing migration of NSCLC cancer cells (see Fig. 8). Our data provide more evidence to consider K_Ca_3.1 channels as a therapeutic target in NSCLC, especially in advanced stages of tumor spreading and EGFR-TKI resistance. Importantly, safety of senicapoc was evidenced through a phase III clinical trial conducted for sickle cell disease and its repurposing for a phase II clinical trial for Alzheimer’s disease [37–39].

## Methods

### Cell culture

All NSCLC cell were kept at 37 °C in a humidified atmosphere containing 5% CO_2_. The highly aggressive A549-3R lung adenocarcinoma [40] cells were cultured in DMEM (Sigma-Aldrich, USA) supplemented with 10% fetal calf serum (FCS, Sigma Aldrich). H1975 cells, obtained from American Type Culture Collection were cultured in RPMI1640 supplemented with 10% FCS. H1975 cells are erlotinib-resistant because of a T790M mutation of the EGFR. Lastly, we obtained A549-3R cells that are partially resistant to erlotinib (A549res) as described in [17].

### Bioinformatic correlation analysis

We used the *Gene Expression Omnibus* database (GEO; http://www.ncbi.nlm.nih.gov/geo) to compare microarray expression data of human erlotinib-sensitive with erlotinib-resistant NSCLC cell lines. The raw data of three data sets (GSE31625, GSE38310, GSE38404) were processed using the Bioconductor package in R (R Core team). We correlated the expression value of the *KCNN4* gene (coding for K_Ca_3.1 channels) with those of all the other genes present in the datasets by applying the Spearman correlation method. A cut-off of the correlation value (COR > 0.9) was applied. To create a short list of genes, a comparison was made between the highly correlated genes of the 3 datasets using the online software Venny 2.0. We performed a gene ontology analysis of the selected highly correlated genes using the online tool PANTHER (protein analysis through evolutionary relationships) version 11.1.

### Caspase 3 activation measurements

We measured the activation of caspase 3 in living cells after 24 h treatment using the CaspGLOW^TM^ Red Active Caspase-3 staining kit (BioVision Inc., USA). For each condition 3 × 10^5^ cells were seeded in a glass-bottom dish (pre-coated at 37°C with 0.1% poly-L-lysin for 30 min) and cultured overnight; the day after, individual dishes were subjected to different treatments. After 24 h of treatment, the cells were washed with PBS and then stained with Red-DEVD-FMK at 37 °C in the dark for 1 h and further processed according to the manufacturer’s protocol. Cells were kept in 500 μL of the washing buffer and fluorescent images were recorded (546 nm excitation, 590 nm emission). Images from ten fields of view for each dish were acquired using a ZEISS microscope Axiovert 200 with a digital camera (Model 9.0, RT-SE-Spot, Visitron Systems, Germany) controlled by MetaVue software (Visitron). Cellular mean fluorecence intensities were determined with ImageJ.

### Isolation of mitochondrial proteins

Mitochondria from human NSCLC adenocarcinoma tissue samples were isolated by suspending ~2 g of the tissue in 1.5 mL TES buffer (300 mM sucrose, 10 mM TES, 0.5 mM EGTA, pH 7.4). Suspended cells were fragmented with a Dounce homogenizer. Intact cells were pelleted by centrifugation at 1000 × *g* at 4 °C for 10 min, resuspended in TES buffer, homogenized and centrifuged again. The supernatants were pooled (200 µL aliquots were stored at −80 °C as “fraction 1”) and centrifuged at 6000 × *g* for 10 min at 4 °C. The resulting pellet was resuspended in 300 µl TES buffer (50 µL aliquots were stored at −80 °C as “fraction 2”). The remaining material was further purified on a Percoll gradient (60%, 30%, and 18% in TES buffer) by centrifugation at 8500 × *g* at 4 °C for 10 min. The floating material in the 18% layer contains ER and plasma membrane contaminants (denominated “fraction 3”). The upper interface contains purified mitochondria (denominated “m1”). Both fractions were collected and washed three times in TES buffer with centrifugation at 17,000 × *g* at 4 °C for 10 min.

We evaluated cytochrome C release from mitochondria to the cytosol of A549, A549res or H1975 cells that were grown in 100 mm tissue culture dishes to 90% confluence. After 5 h treatment, cells were harvested by trypsinization. The cellular pellet was then resuspended in 400 µL of buffer A (20 mM MOPS KOH pH7.4; sucrose 250 mM). 400 µL of buffer A supplemented with 1 mg/mL digitonin (#D5628, Sigma Aldrich) was added to the solution which was then centrifuged at 5.000 x g at 4 °C for 3 min. The supernatant contained the cytosolic fraction and was stored at -80°C, while the pellet was resuspended in 600 µL of buffer B (20 mM MOPS KOH pH 7.4; sucrose 250 mM; EDTA-N_4_ 1 mM) for isolation of the mitochondrial fraction. After 5 min on ice the solution was centrifuged at 10.000 x g at 4 °C for 3 min; the pellet with the mitochondrial fraction was resuspended in buffer A and stored at -80 °C before further analysis.

### Western blot analysis

Cells were lysed in RIPA buffer buffer (50 mM Tris, 150 mM NaCl, 0.1% SDS, 0.5% sodium deoxycholate, 1% NP-40 and protease inhibitors from Roche, Germany). Before gel-loading, the samples were mixed with a 5x SDS-Page sample buffer (0.225 M Tris-Cl pH 6.8, 50% glycerol, 5% SDS, 0.25 M DTT, and 0.05% bromphenol blue) and boiled at 95 °C for 5 min. After electrophoresis, proteins were transferred to a nitrocellulose membrane (Millipore, USA) using a Trans-blot^TM^ SD (BioRad, USA). The membrane was blocked in Tris-buffered saline (TBS containing 5% skim milk and 0.05% Tween) for 1 h. We used the following antibodies: rabbit anti-K_Ca_3.1 (1:500, #AV35098, Sigma-Aldrich), rabbit anti-BAK (1:200, #AHP2279, Bio-Rad), mouse anti α-tubulin (1:5000, #6199, Sigma-Aldrich), rabbit anti-vinculin (1:10.000, V9264, Sigma Aldrich), rabbit anti-ATP5a (1:500, AB14748, Abcam, UK) and rabbit anti-cytochrome C (1:300, 556433, BD Biosciences, USA), rabbit anti-β1 integrin (1:10,000, #AB1952, Millipore), rabbit anti-SAPK/JNK (1:500, #9252, Cell Signaling, USA), rabbit anti-Phospo-SAPK/JNK (1:200, #9251, Cell Signaling), rabbit anti-c-Jun (1:500, #9165, Cell Signaling), rabbit anti-Phospo-c-Jun (1:200, #91952, Cell Signaling) and rabbit anti-GAPDH (1:5000, #6199, Sigma-Aldrich) as primary antibodies, goat anti-mouse and goat anti-rabbit (1:10,000, Sigma-Aldrich) as secondary antibodies. Clarity Western ECL Substrate (BioRad) was used as chemiluminescence substrate. Signals were revealed using the ChemiDoc Gel Imaging System (BioRad) and quantified with Image Lab software (BioRad). Cytochrome C release into the cytoplasm is quantified as the ratio of cytoplasmic cytochrome C expression (cytochrome C band intensity normalized to that of the cytoplasmic marker vinculin) and mitochondrial cytochrome C expression (cytochrome C band intensity normalized to that of the mitochondrial marker ATP5a).

### Mitochondrial membrane potential measurements

The mitochondrial membrane potential was measured using the cationic fluorescent dye Tetramethylrhodamine Methyl Ester (TMRM; #T668, ThermoFisher, USA). We seeded 7 × 10^4^ A549, A549res or H1975 cells in glass coverslips (pre-coated with 0.1% poly-L-lysine at room temperature for 30 min) in 35 mm cell culture dishes and cultured overnight. On the following day, cells were incubated with 25 nM TMRM in HBSS at 37°C in the dark. After 20 min the TMRM concentration was reduced to 5 nM, and 4 µM cyclosporine H were added. The glass coverslips were placed in an environmental chamber (37 °C) on the stage of a Leica SP5 confocal microscope and images were acquired in 5 min intervals. The recording started with a 10 min control period. Thereafter, compounds were added at the indicated concentrations and images of the same visual field were taken in 5 min intervals for another 30 min. At the end of each experiment, 1 μM of the mitochondrial oxidative phosphorylation uncoupler, FCCP (carbonyl cyanide-p-trifluoromethoxyphenylhydrazone), was added and additional images were taken in 2.5 min intervals for a period of 5 min.

### Superoxide release measurements

We seeded 7 × 10^4^ A549, A549res or H1975 cells in glass-bottom 96-well cell imaging plates in their culture medium and incubated them overnight. The day after the medium was replaced with 100 µL HBSS solution containing 5 µM MitoSOX™ Red (#M36008, Thermo Fisher Scientific) and 2 nM Höchst 33342 (for nuclei staining; Thermo Fisher) and kept at 37 °C in the dark for 30 min. Thereafter, the HBSS solution was replaced with 100 µL HBSS without fluorescent dyes. The 96-well plate was placed on the Operetta CLS™ (PerkinElmer), and fluorescent images were acquired in 5 fields of view for each well in 5 min intervals. After 10 min, treatment compounds were added and cells were imaged every other 5 min for 50 min (each condition in duplicate). The acquired time-lapse stacks were processed and analyzed using Harmony^®^ 4.8 high-content analysis software (PerkinElmer, USA).

### Real-time ATP production assay

Total, mitochondrial (mitoATP) and glycolytic (glycoATP) ATP production rates, respectively, were indirectly determined in real time using the XF Real-Time ATP Rate Assay Kit with a Seahorse extracellular flux (XF) HS Mini Analyzer. The oxygen consumption rate (OCR) was quantified and ATP rates were calculated according to the manufacturer´s protocol. We seeded 1.5 × 10^4^ A549, A549res or H1975 cells in standard culture medium and incubated at 37°C for 24 h. The day after the medium was replaced with Seahorse XF DMEM Medium, pH 7.4 with 10 mM of XF glucose, 1 mM of XF pyruvate, 2 mM of XF glutamine. OCR was measured at pre-set time intervals upon the automated additions of compounds (DMSO (1:1000), 10 µM senicapoc, 1.5 µM of oligomycin and at the end 0.5 µM rotenone + 0.5 µM antimycin A).

### Cell- matrix adhesion assay with single cell force spectroscopy

Single cell force spectroscopy (SCFS) was performed with an atomic force microscope (AFM) in a similar way as described previously [16]. Cells were cultivated for 1 day and then treated with the desired compounds for 24 h. At the same time half of a 40 mm tissue culture dish (Techno Plastic Products AG) was coated with 100 μL of an ECM-like matrix of the following composition: RPMI (10.4 g/L), HEPES (10 mM), laminin (40 μg/mL; Sigma Aldrich), fibronectin (40 μg/μL, Becton Dickinson), collagen IV (5.4 μg/μL; Corning, USA), collagen III (12 μg/μL, Corning), rat tail collagen I (800 μg/mL, R&D system, USA) and H_2_O, adjusted to pH 7.4 with 1 M NaOH. For matrix polymerization the coated dish was left overnight at 37 °C and 5% CO_2_. On the day of the experiment cells were trypsinized (0.25% trypsin), resuspended in medium and left on ice until the experiment. At the same time, the other half of the 40 mm dish was coated with 0.5% gelatin at 37 °C for 30 min. Thereafter, the dish was washed with PBS and filled with 1 mL of Ringer’s solution (NaCl 122.5 mM, KCl 5.5 mM, glucose 5.5 mM, CaCl_2_ 1.2 mM, MgCl_2_ 0.8 mM, HEPES 10 mM, adjusted to pH 7.4 with 1 M NaOH). It was placed on the heated stage (37°C) of a ZEISS microscope Axiovert 135 M equipped with the CellHesion^®^ 200 module (JPK Instruments, Germany).

Prior to all experiments, a tipless cantilever (#ARROW-TL1-50, NanoWorld AG) was incubated in PBS containing 1 mg/ml wheat germ agglutinin for 20 min (WGA; #L-9640, Sigma-Aldrich). The cantilever was then placed on the CellHesion^®^ 200 module (AFM head) and placed over the 40 mm dish without cells for calibration. Spring constant (between 0.015 N/m and 0.06 N/m) and sensitivity were obtained by the CellHesion^®^ software (JPK, SPM, version 4).

After calibration of the cantilever 10 µL of the cell suspension were seeded in the dish and a single cell was picked from the gelatine covered area of the dish with the WGA-coated cantilever (DFK31BF03, ImagingSource, Germany). The cantilever always approached a cell with a contact time of 10 s and a setpoint force of 2 nN, using a pulling length (z-length) of 100 µm and a velocity during approach and retraction of 5 µm/s. Thereafter, the cantilever with the bound cell was moved to the ECM-like matrix area. The bound cell was lowered onto the ECM-like matrix for 2 s with a setpoint force of 1nN. Then the cell was lifted back using the same pulling length and velocity values as for the picking procedure. The interaction forces between the cells and the matrix were recorded by CellHesion^®^ software in a force-distance curve. For each experiment the attached cell was brought into contact with the ECM-like matrix for at least 5 times at five different positions. The maximal adhesion force (nN) was derived from the force-distance curves using the JPK Data Processing software (version 4.3.18).

### Cell migration in a 3D matrix

Cell migration was recorded in a 3D matrix using an Ibidi μ-Slide I Luer. Cells were suspended at a final density of 1 × 10^6^ cells/mL and supplemented with the desired treatment compounds at the indicated concentration. Then cells were mixed in a ratio of 1:4 with an ECM-like matrix of the following composition: RPMI (10.4 g/L), HEPES (10 mM), laminin (20 μg/mL; Sigma Aldrich), fibronectin (40 μg/μL, Becton Dickinson), collagen IV (5.4 μg/μL; Corning), collagen III (12 μg/μL, Corning), rat tail collagen I (1.600 μg/mL, R&D system) and H_2_O adjusted to pH 7.4 with 1 M NaOH. A final volume of 200 μL of the matrix/NSCLC cell suspension was loaded into an Ibidi μ-Slide I Luer and let polymerize at 37°C for 3 h (with 10 ng/mL EGF (Sigma Aldrich)). After polymerization, the migration experiment started. The focal plane was set in the middle of the μ-Slide. Images were acquired in 10 min intervals for 12 h using a ZEISS microscope Axiovert 40 C (Carl Zeiss, Germany), linked to a MicroCam SP 3.1 video camera (Bresser, Germany) controlled by the MicroCamLab software (Bresser). Migration of NSCLC cells was analyzed with Amira^®^ software (ThermoFisher). Using a self-made JAVA program the positions of the centroids were determined for each time point. Migration speed and translocation were calculated as shown by Glaser et al. 2021 [17].

### Statistical analysis

All data are shown as mean ± SEM. We used a two-tailed or multiple Student’s *t*-test for comparison between two groups. Mann–Whitney *U*-test was used for not normally distributed data. For comparing >2 groups we applied Ordinary 1-way ANOVA or Ordinary 2-way ANOVA, followed by Tukey’s or Bonferroni post hoc test. In both cases *p*-values < 0.05 were considered as statistically significant.

### Supplementary information

Supplemental material figures

Original Data File

## Data Availability

All relevant data are available from the authors upon request.
