# Supplementary material for: Targeting KCa3.1 channels to overcome erlotinib resistance in non-small cell lung cancer cells
Source: Cell Death Discov. 2024 Jan 4;10:2. doi: 10.1038/s41420-023-01776-5 (PMC10767088; doi:10.1038/s41420-023-01776-5)
Supplement: Supplementary file 1 — Supplemental material figures [file 41420_2023_1776_MOESM1_ESM.pdf]

Supplementary 1

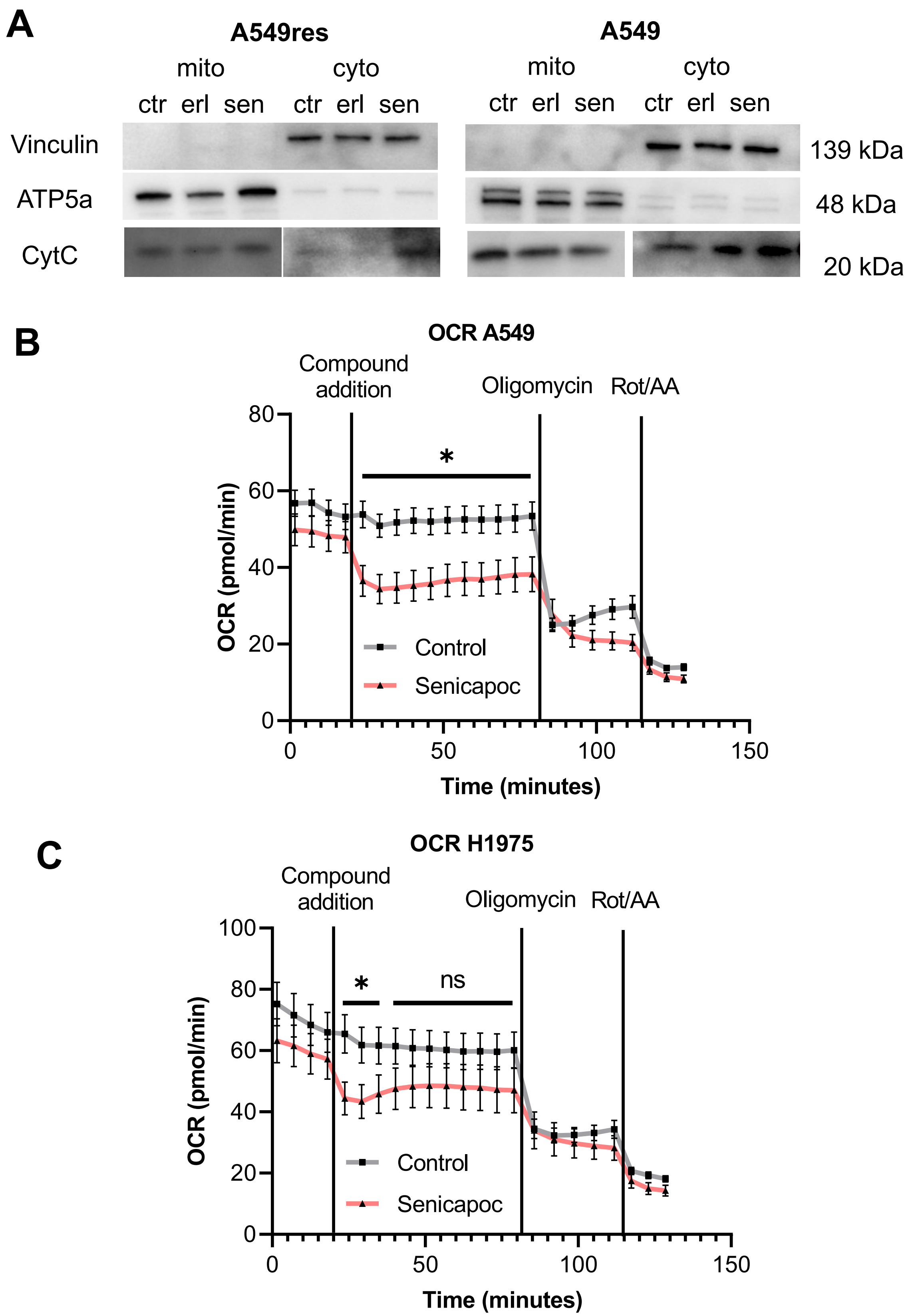

Supplementary 1. A. Western blots used to quantify cytochrome C release into the cytosol of A549 and A549res cells. Vinculin (139 kDa) and ATP5a (48 kDa) served as cytosolic and mitochondrial markers, respectively. **B-C.** Oxygen consumption rate (OCR) of A549 **(B)** and H1975 cells **(C)** under control conditions (DMSO 1:1,000) and following the application of senicapoc (30 µM). After 79 min oligomycin A was added and after 112 min Rot/AA, a mix of antimycin A (AA) and rotenone (Rot), was added. *: $p < 0.05$; multiple t-test between each time point.

Supplementary 2

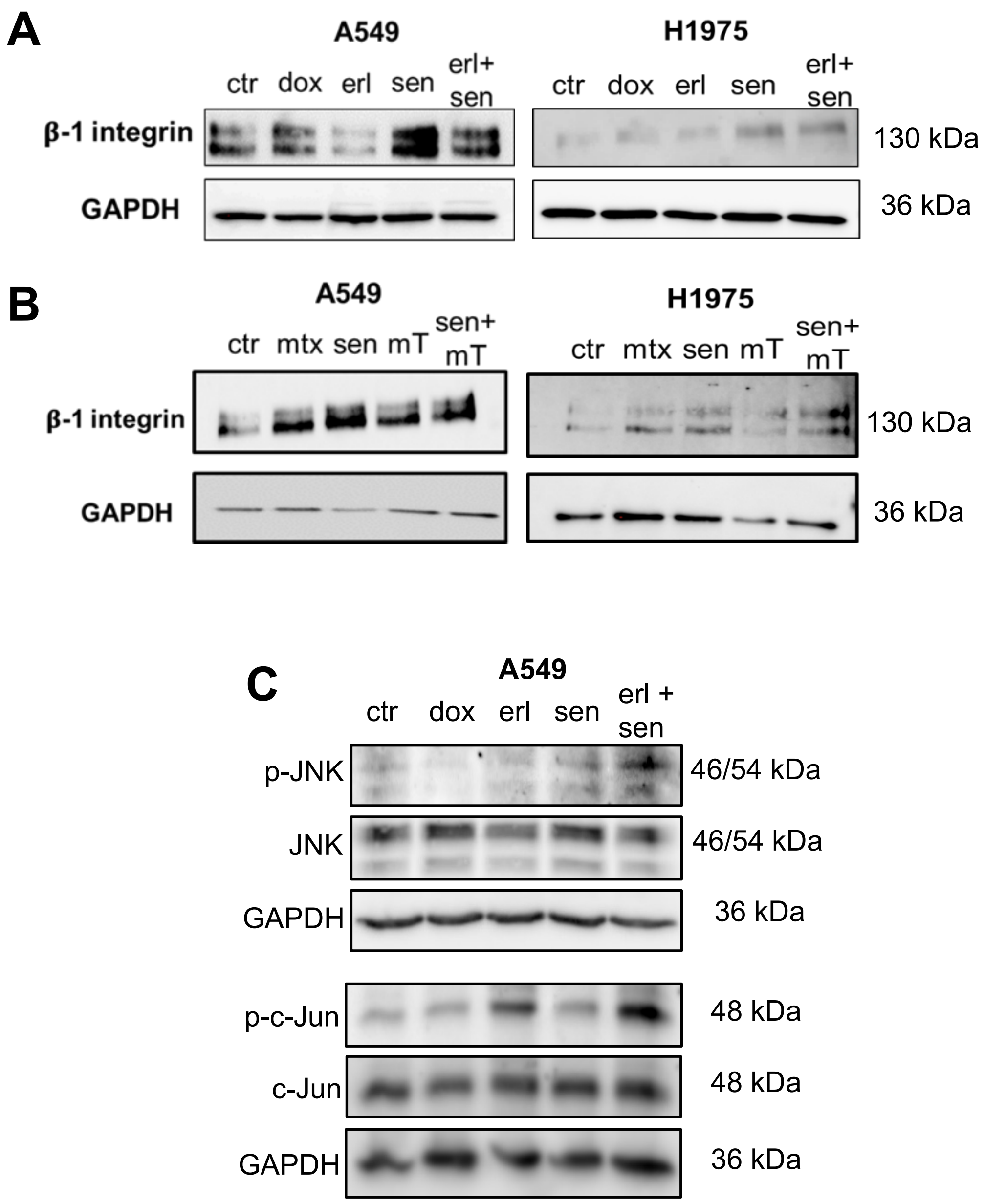

Supplementary 2. A. Western blots showing the regulation of β1-integrin expression in A549 and H1975. Protein lysates were obtained after 24h pretreatment (ctr = DMSO 1:1000; erl = erlotinib 10 μM; erl+sen = erlotinib 10 μM + senicapoc 30 μM; sen = senicapoc 30 μM; dox = doxorubicin 100nM). **B.** Western blots showing the regulation of β1-integrin expression in A549 and H1975. Protein lysates were obtained after 24h pretreatment (ctr = DMSO 1:1000; erl = erlotinib 10 μM; erl+sen = erlotinib 10 μM + senicapoc 30 μM; sen = senicapoc 30 μM; dox = doxorubicin 100nM). **C.** Western blots to illustrate phosphorylation of JUN and JNK in A549 cells under 5 different conditions. Protein lysates were obtained after 24 h pretreatment (ctr = DMSO 1:1000; erl = erlotinib 10 μM; erl+sen = erlotinib 10 μM + senicapoc 30 μM; sen = senicapoc 30 μM; dox = doxorubicin 100nM).
