# Supplementary material for: Targeting KCa3.1 channels to overcome erlotinib resistance in non-small cell lung cancer cells
Source: Cell Death Discov. 2024 Jan 4;10:2. doi: 10.1038/s41420-023-01776-5 (PMC10767088; doi:10.1038/s41420-023-01776-5)

Original blots

Figure 2C

CytC

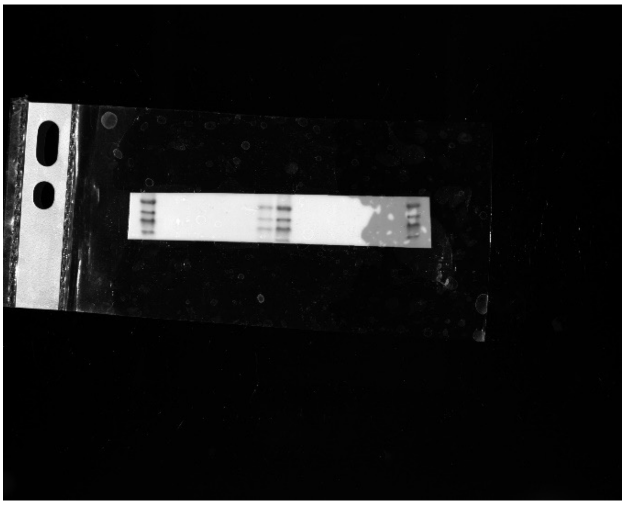

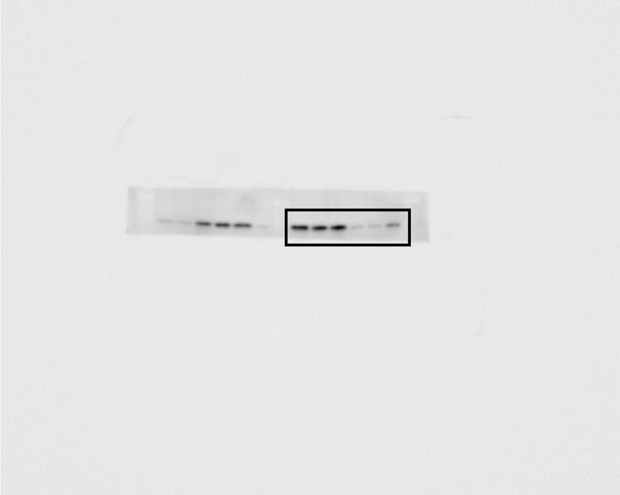

ATP5a

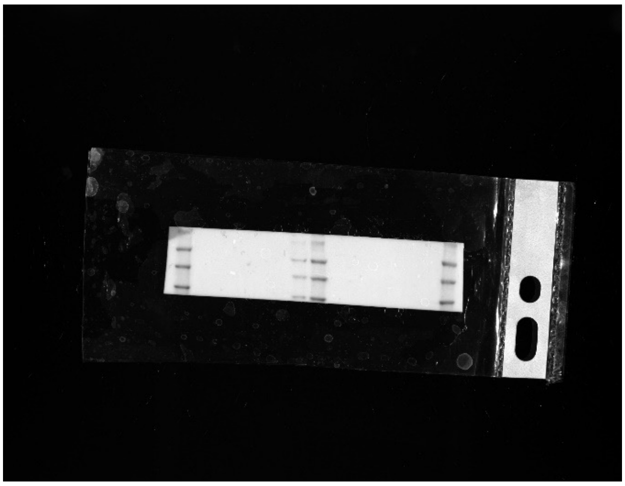

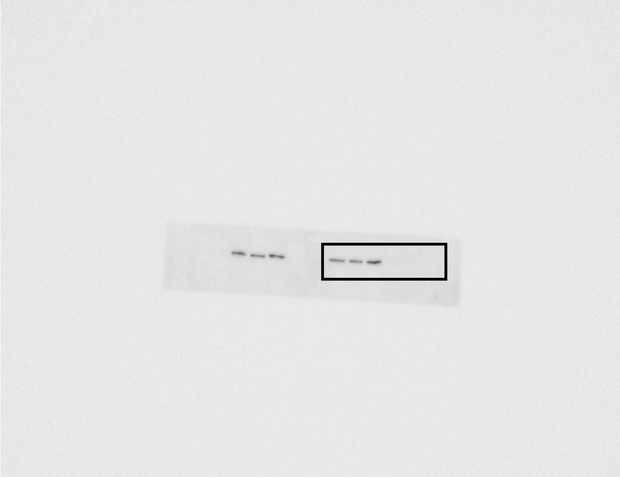

Vinculin

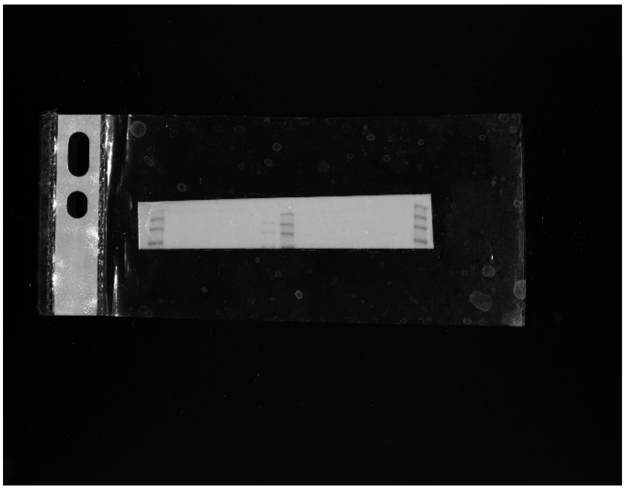

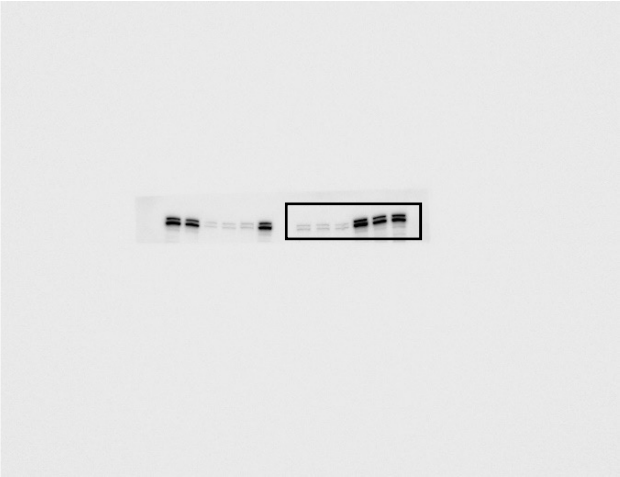

Figure 2E

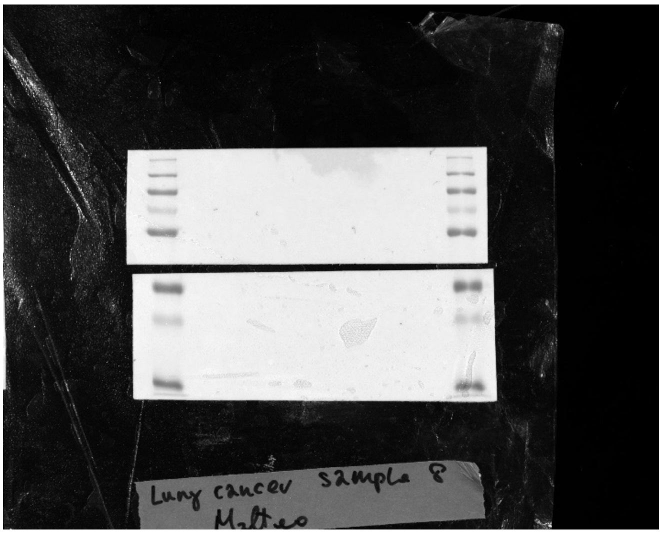

$K_{Ca}3.1$

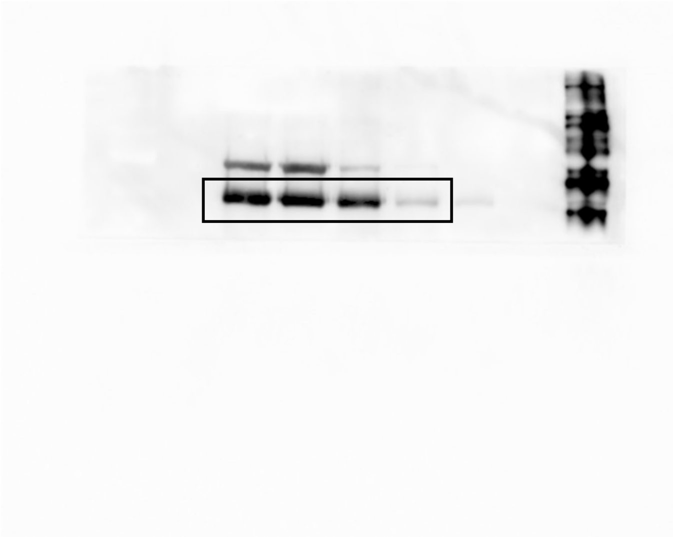

BAK

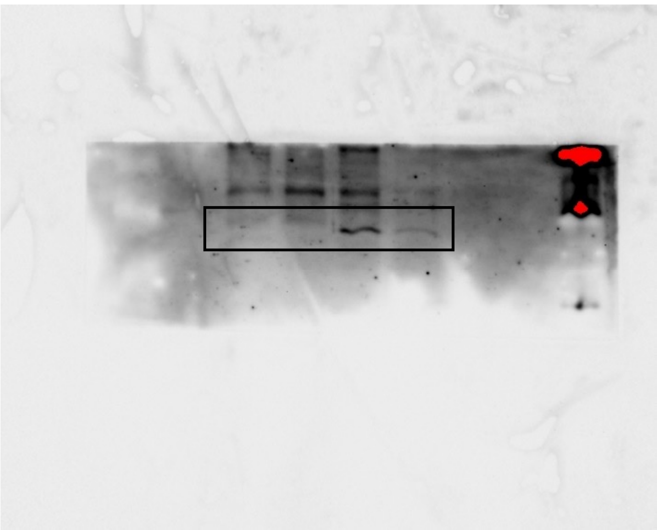

α-tubulin

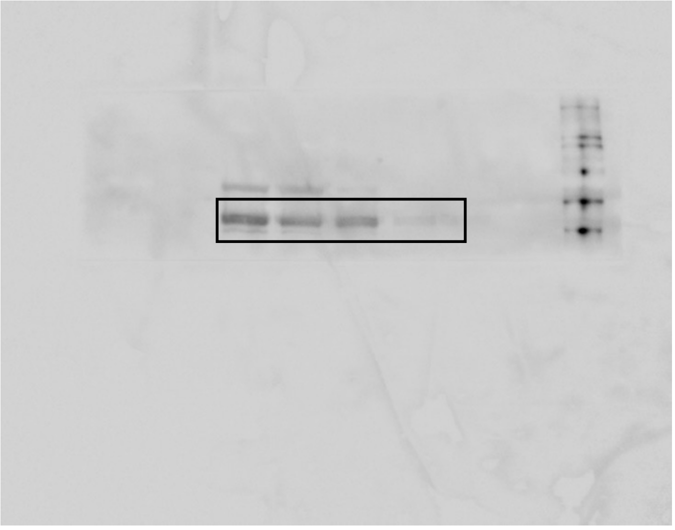

Figure 6A

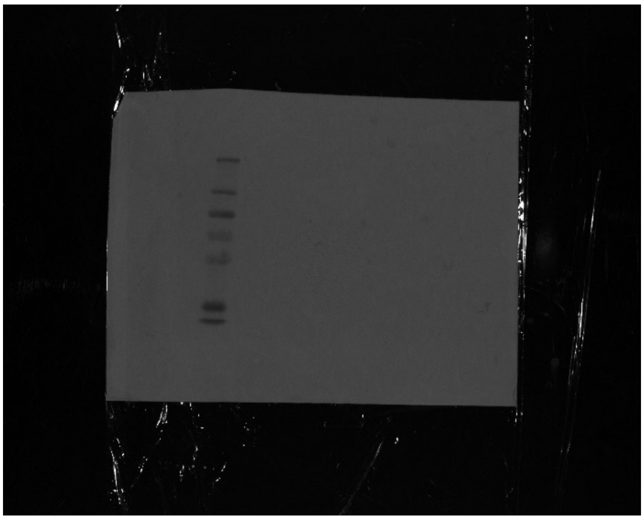

β1-integrin

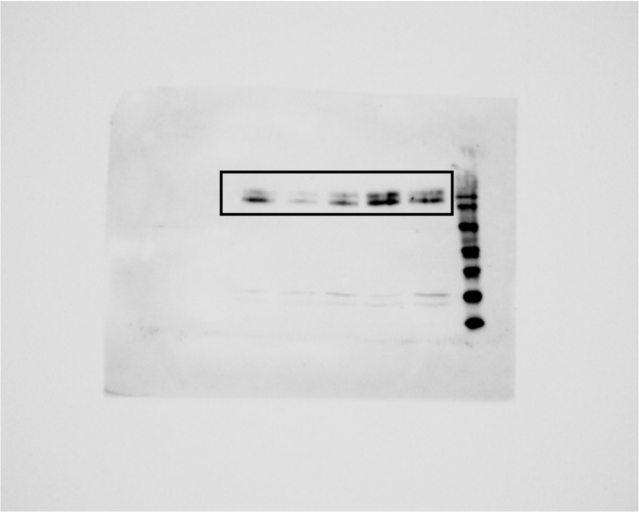

GAPDH

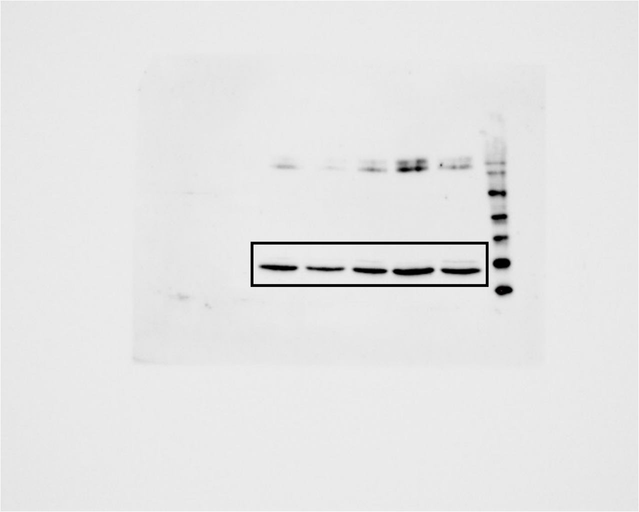

Figure 6F

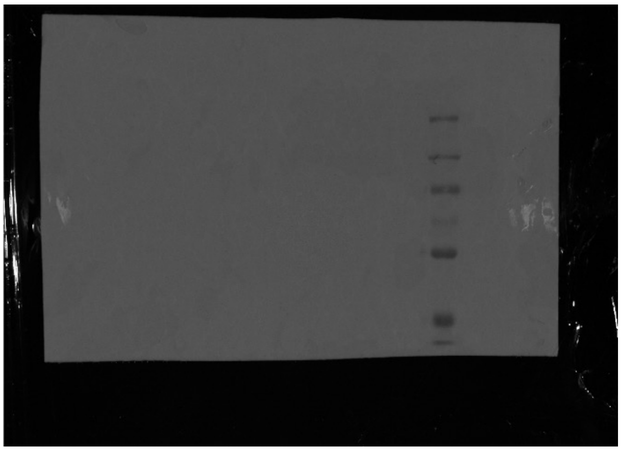

β1-integrin

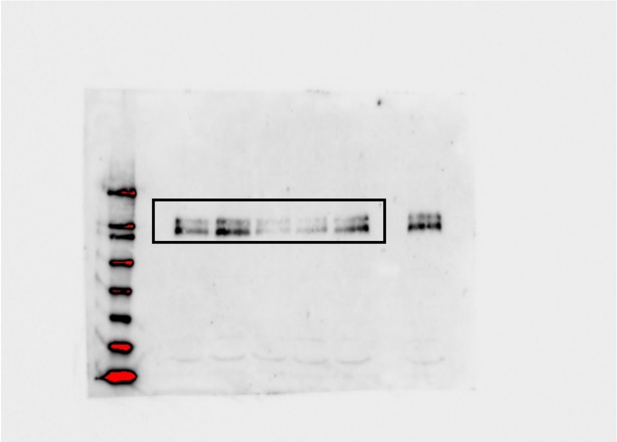

GAPDH

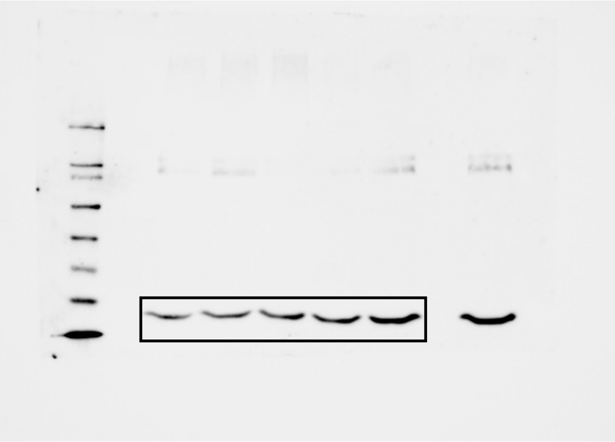

Figure 7B

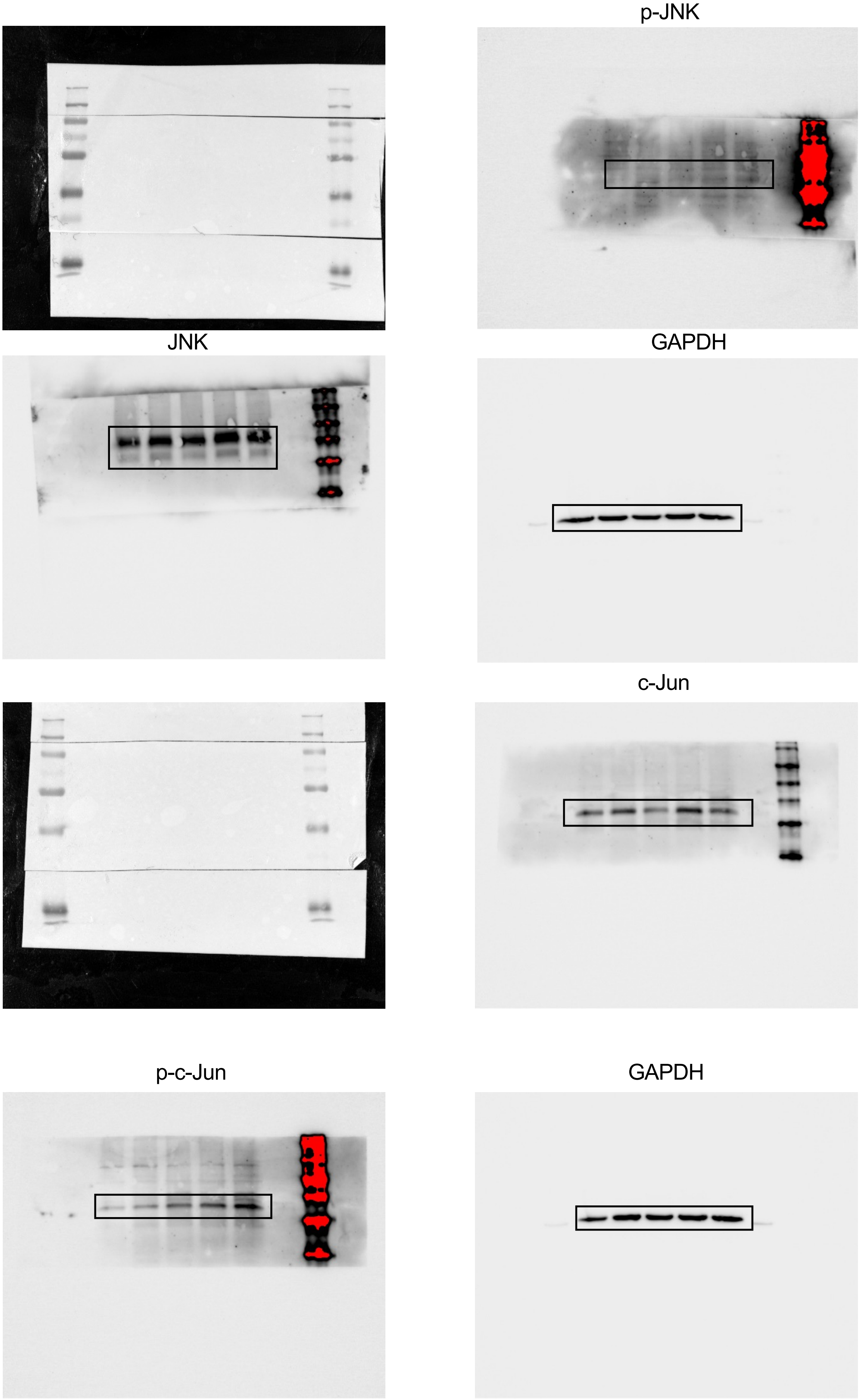

Figure 7D

A549

β1-integrin

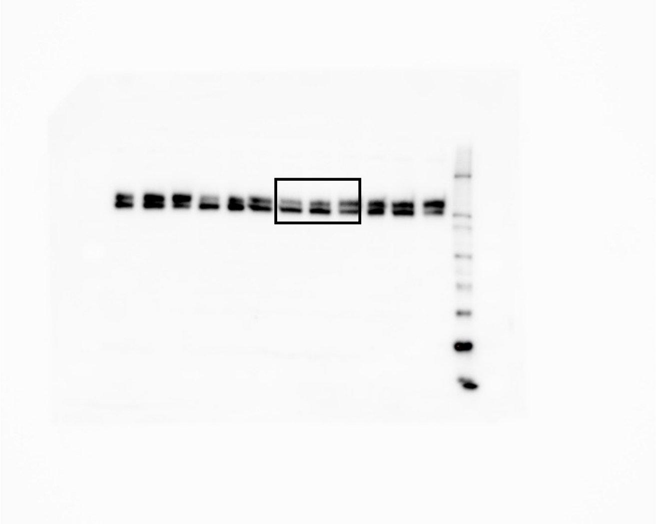

GAPDH

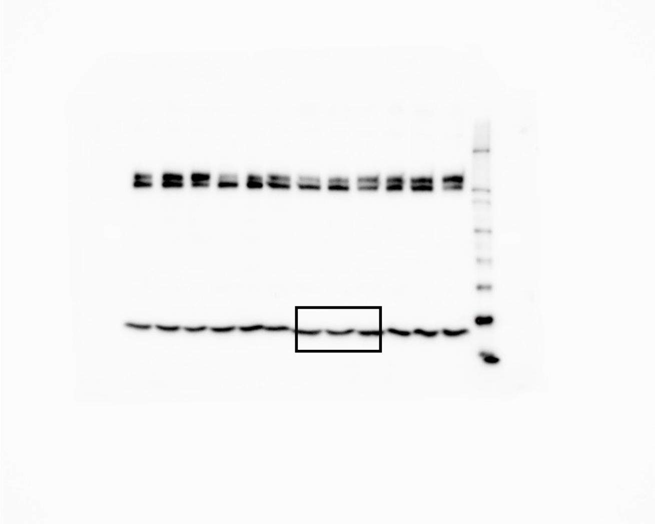

H1975

β1-integrin

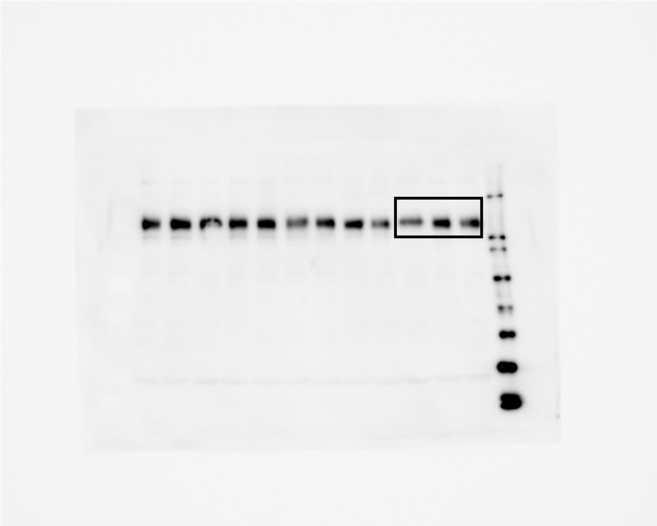

GAPDH

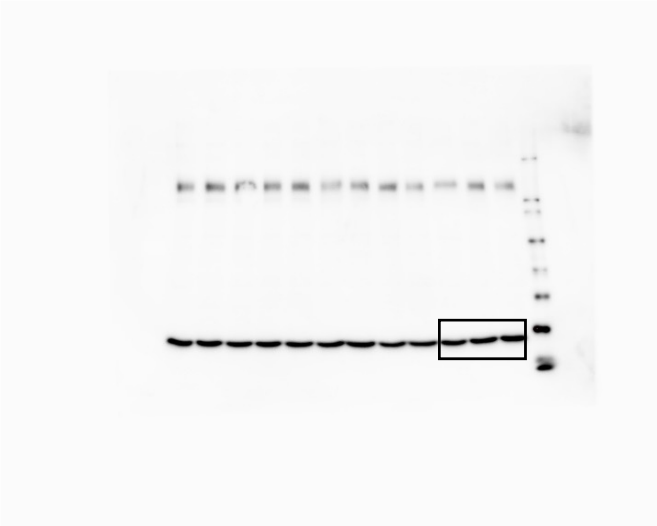

Supplementary 1A

CytC

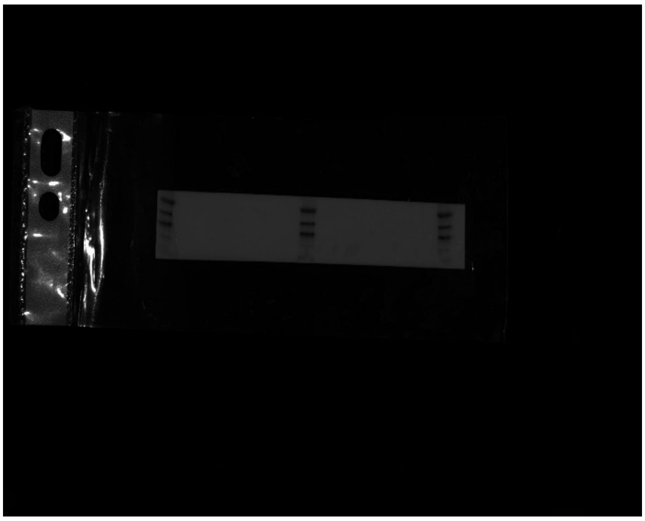

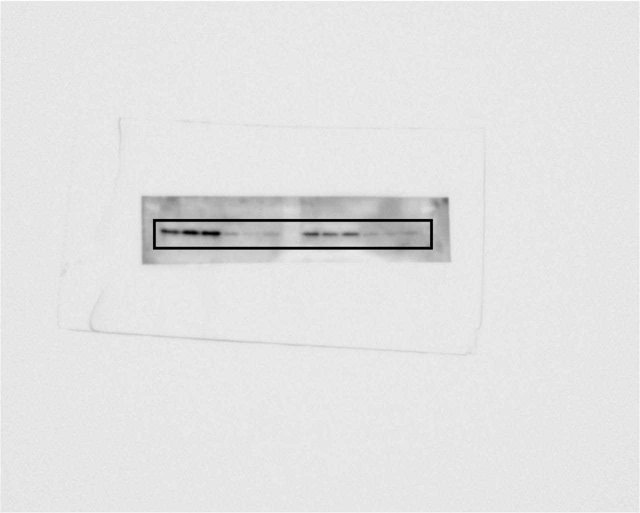

ATP5a

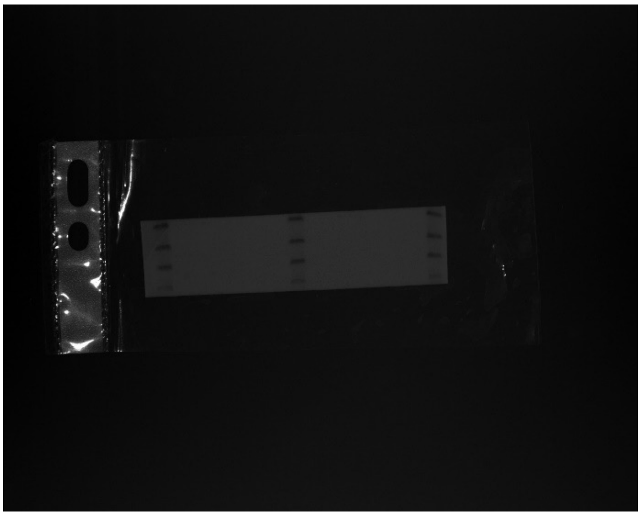

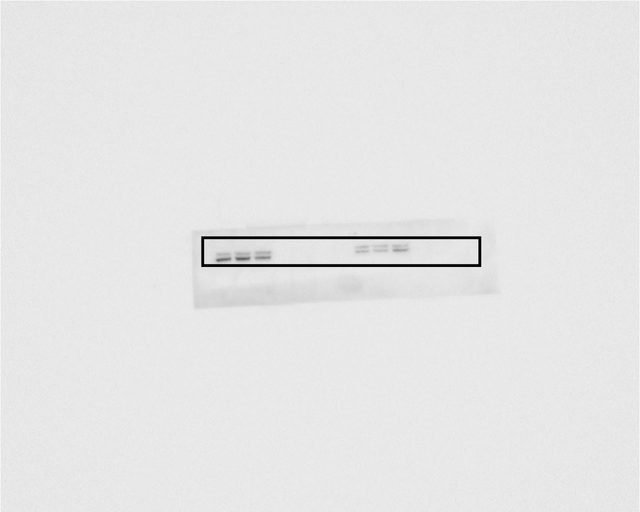

Vinculin

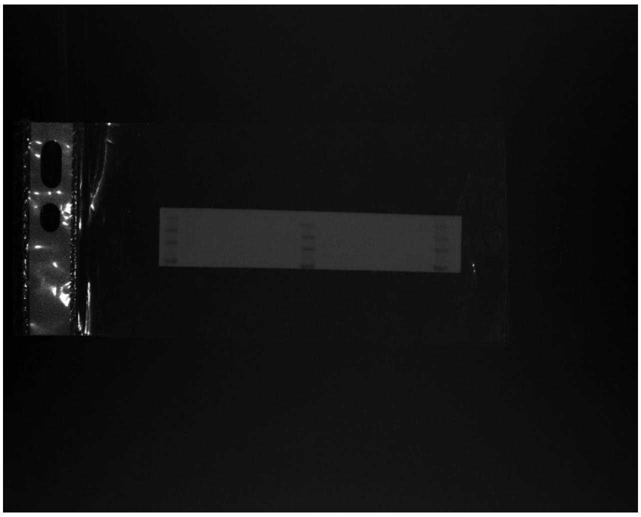

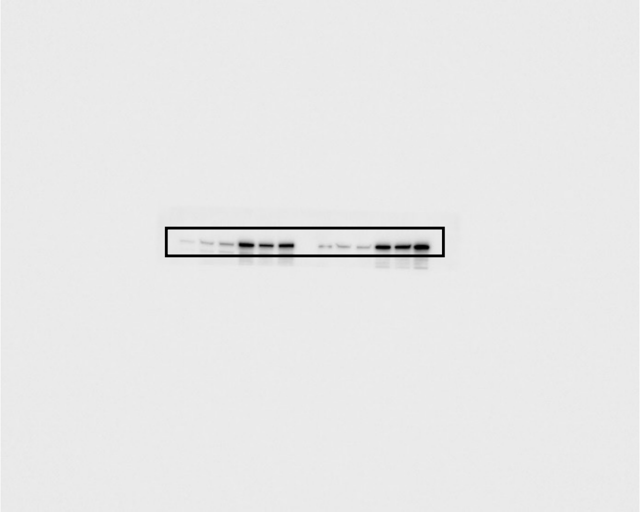

Supplementary 2A

A549

β1-integrin

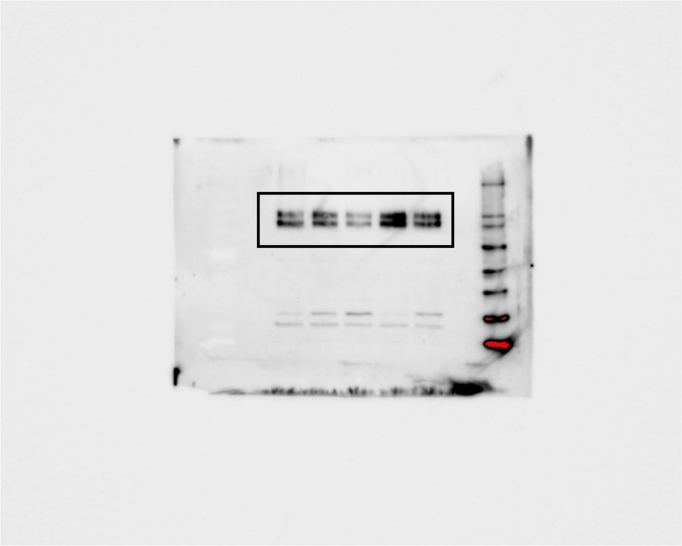

GAPDH

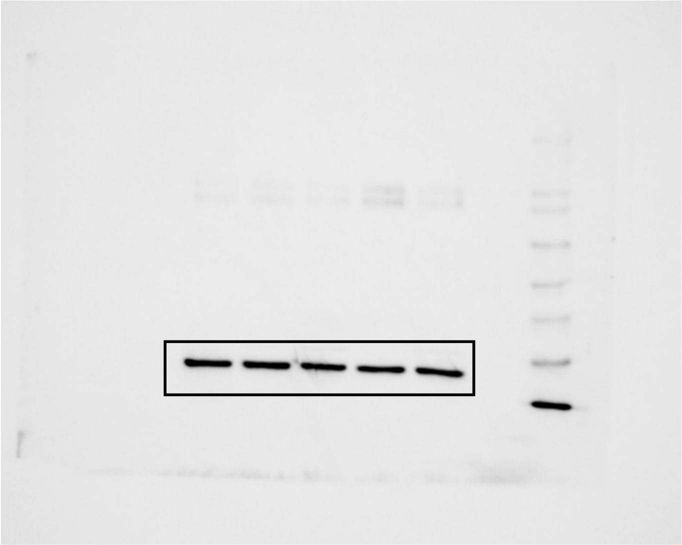

H1975

β1-integrin

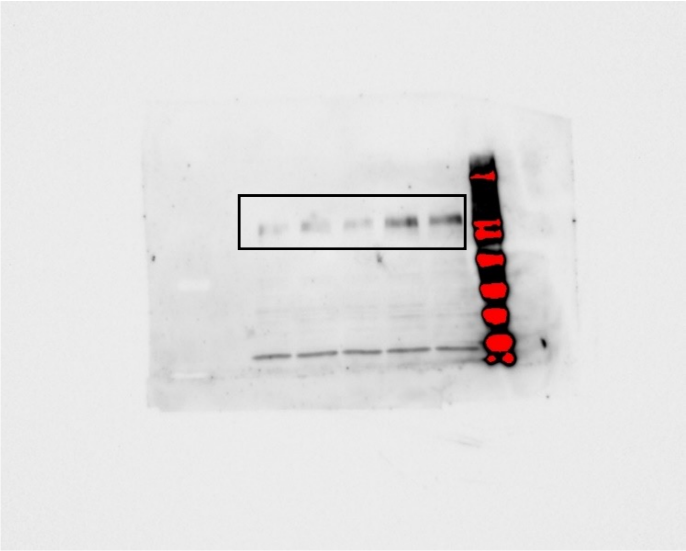

GAPDH

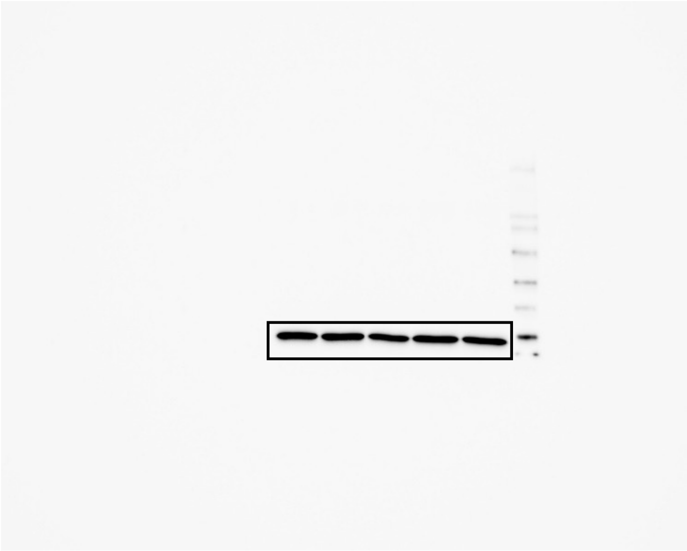

Supplementary 2B

A549

β1-integrin

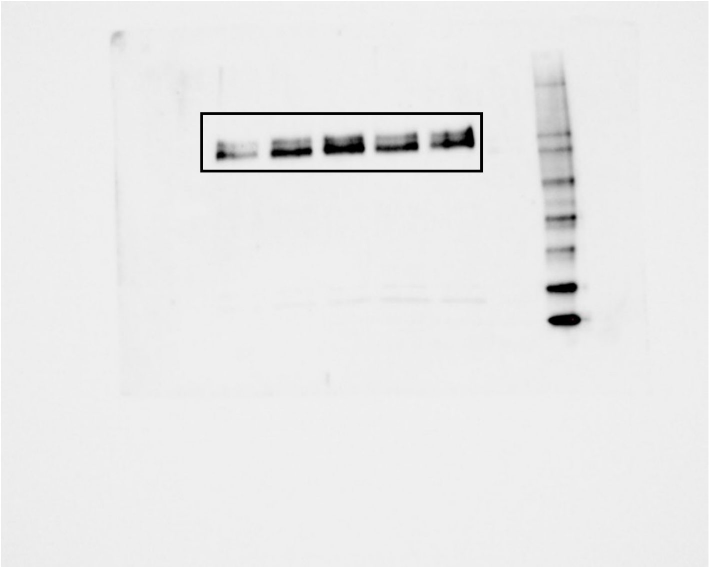

GAPDH

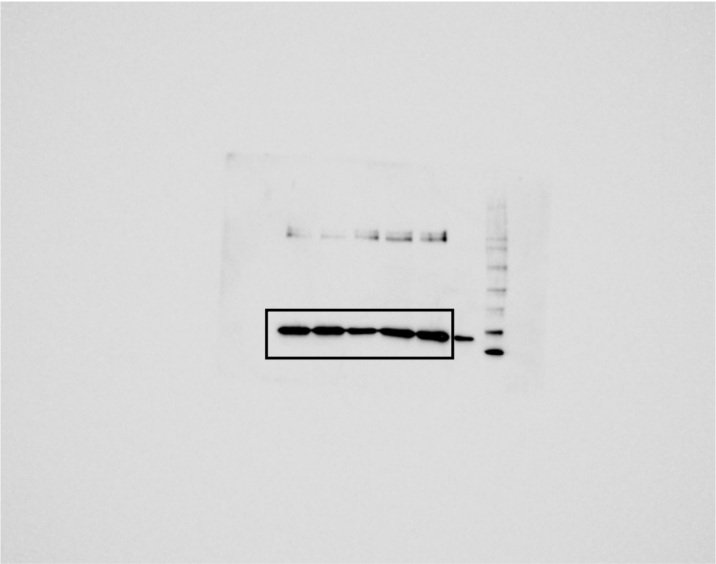

H1975

β1-integrin

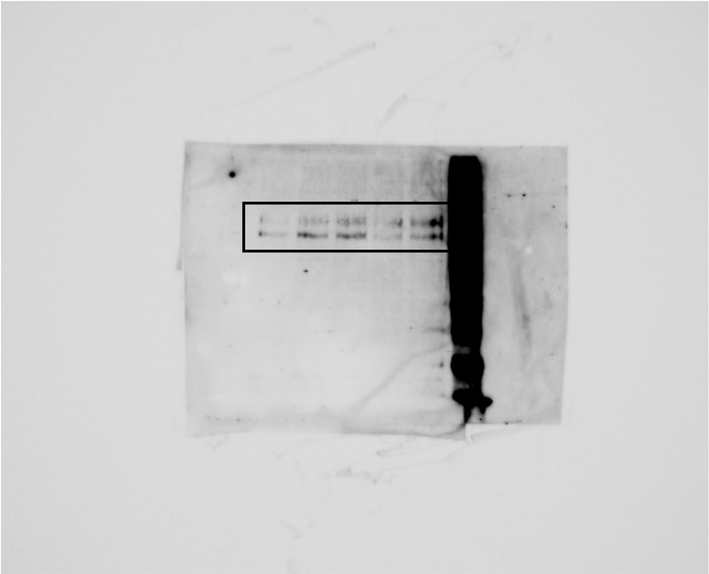

GAPDH

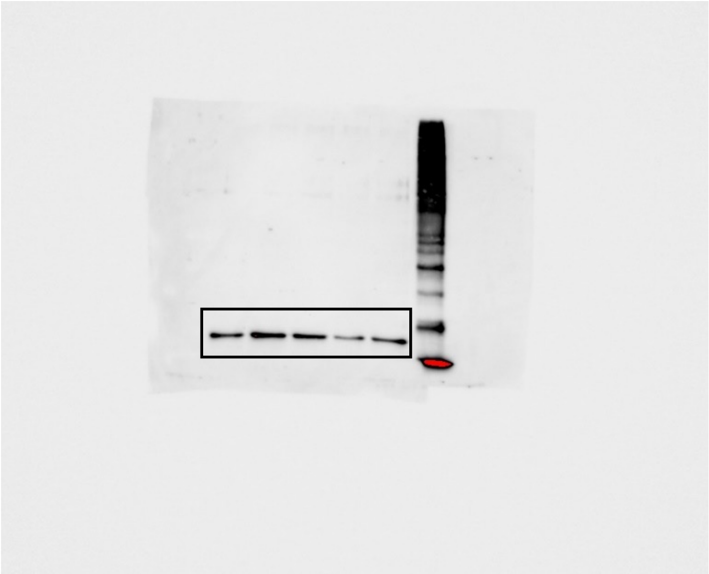

Supplementary 2C

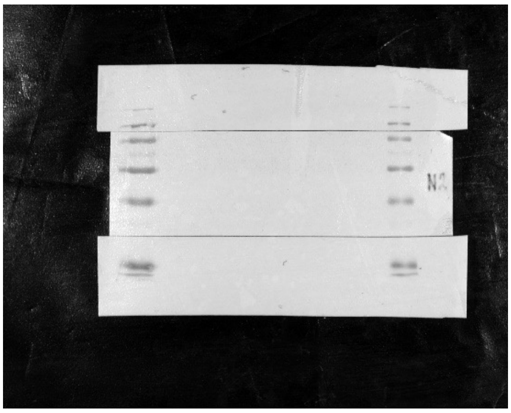

p-JNK

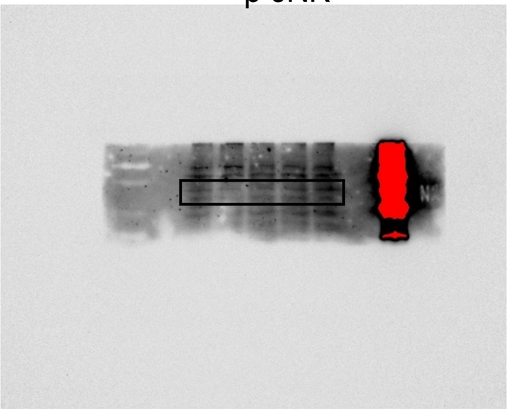

JNK

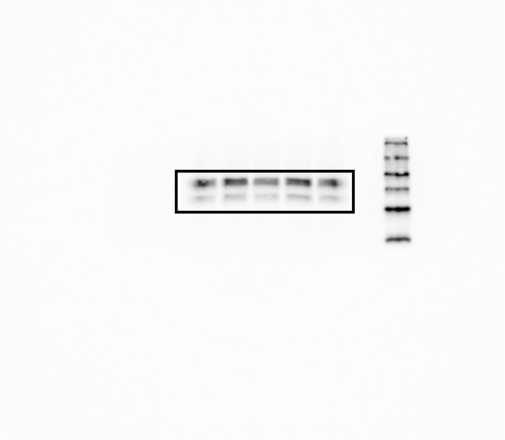

GAPDH

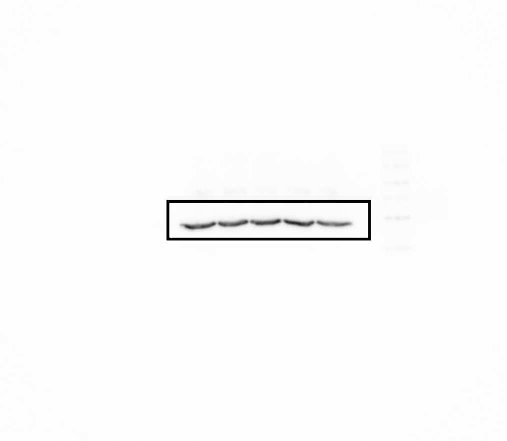

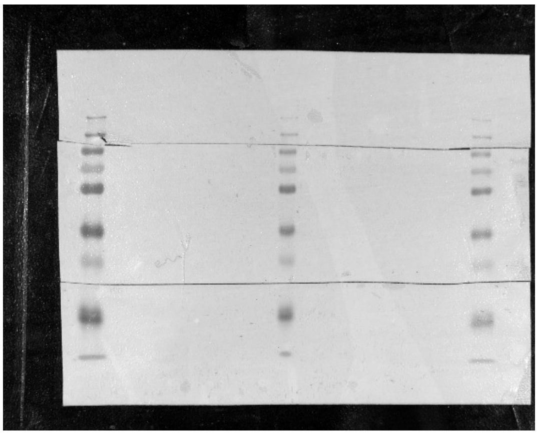

c-Jun

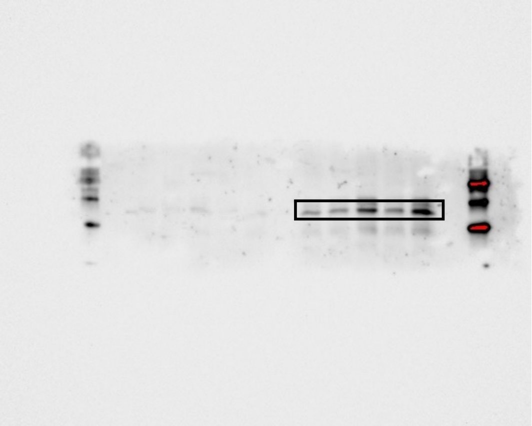

p-c-Jun

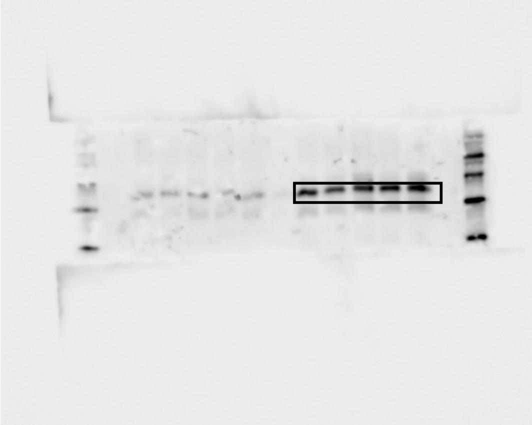

GAPDH

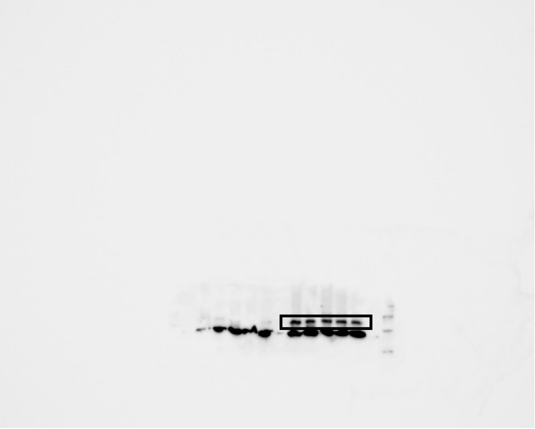

Supplement: Supplementary file 2 — Original Data File [file 41420_2023_1776_MOESM2_ESM.pdf]
